# Directional differences in saccades

**DOI:** 10.3389/fneur.2026.1945647

**Published:** 2026-10-23

**Authors:** Takashi Jono, Koji Takahashi, Hiroya Hara, Yosuke Kudo, Erich Schneider, Mitsuo Shiono, Fumiaki Tanaka, Ken Johkura

**Affiliations:** 1 Department of Neurology, Yokohama Brain and Spine Center, Yokohama, Japan; 2 Department of Neurology and Stroke Medicine, Yokohama City University Graduate School of Medicine, Yokohama, Japan; 3 Department of Clinical Laboratory, Yokohama Brain and Spine Center, Yokohama, Japan; 4 Brandenburg University of Technology Cottbus, Senftenberg, Germany; 5 Zero C Seven Inc., Tokyo, Japan

**Keywords:** abduction-adduction asymmetries, centrifugal-centripetal asymmetries, horizontal saccade, periocular tissue, vertical saccade

## Abstract

**Introduction:**

Though saccades are believed to be generated by similar neural mechanisms in all directions, measurements show directional differences in movement characteristics. The mechanisms and causes of these differences remain unclear.

**Methods:**

This study investigated directional differences in saccades in 48 healthy participants (28 males, 20 females; mean age 40.38 years). An EyeSeeCam Sci 2 video-oculography system with a sampling rate of 500 Hz was used to measure centrifugal and centripetal horizontal (5 °, 10 °, 15 °, and 20 °) and vertical (5 °, 10 °, and 15 °) visually guided saccades.

**Results:**

Horizontally, when amplitude was large (15 ° and 20 °), saccades in the medial hemifield were faster than in the lateral hemifield in both centrifugal and centripetal directions. Centripetal saccades were significantly faster than centrifugal ones. Vertically, with large amplitude (15 °), saccades in the upper hemifield were significantly faster than in the lower hemifield in both directions. As horizontally, centripetal saccades were faster than centrifugal ones in the upper hemifield. No clear trends appeared in horizontal saccade latency and amplitude. However, shorter latencies for centripetal saccades and larger amplitudes for upward saccades were noted vertically.

**Disucussion:**

The medial-lateral eye position difference likely relates to saccade-vergence interaction. Other directional saccade asymmetries are attributed to physical properties, like the elastic restoring force in the centripetal direction and viscous resistance from eyelid coverage in the lower eye position, rather than neural mechanisms. Saccade motor characteristics are strongly influenced by periocular tissues.

## 1 Introduction

Saccades are rapid eye movements used to direct attention to an object or shift gaze to a new location. The amplitude and direction of a saccade encoded in the superior colliculus are independent of the initial position of the eye and thus not goal-directed (1). Consequently, saccadic eye movements are generally considered similar in all directions, regardless of the initial eye position. However, actual measurements of saccades have revealed directional differences in movement characteristics (e.g., adduction vs. abduction and centrifugal vs. centripetal).

In the case of centrifugal horizontal saccades, the difference between adduction and abduction has often been studied; however, the results vary depending on the experimental approach. Some studies reported faster peak velocities during adduction (2–4) or abduction (5–8), whereas others found no difference between the two conditions (9). Additionally, some studies have suggested that centripetal saccades are faster than centrifugal saccades, possibly because of the mechanical constraints imposed by periocular tissues, although these studies have been limited in their experimental approaches (1–3, 5–7). Few studies have focused on vertical saccades, and currently, no studies have evaluated the differences in movement characteristics between centrifugal and centripetal vertical saccades.

In this study, we measured the centrifugal and centripetal saccades elicited by visual targets that jumped with various amplitudes in both horizontal and vertical directions under controlled conditions in healthy subjects.

## 2 Materials and methods

### 2.1 Participants

Forty-eight healthy subjects (28 males and 20 females; mean age: 40.38 years, range: 22–74 years) with no history of neurological, psychiatric, or ophthalmological disorders participated in this study. Participants with limitations in ocular motility, including convergence, or abnormalities in ocular alignment were excluded, as were those with insufficient visual acuity to clearly fixate on the visual target due to myopia or other refractive errors. The study was approved by our Institutional Ethics Committee (no. 142200801), and informed consent was obtained from each participant after the purpose and procedures were explained.

### 2.2 Eye movement recordings

Eye movements were recorded binocularly using an EyeSeeCam Sci 2 device (EyeSeeTec, Munich, Germany). The device tracks eye movements using lightweight goggles equipped with two infrared video cameras mounted on top. A half mirror is attached to the front of the goggles, allowing the infrared camera to capture eye movement through the half mirror. Pupil position was detected using the dark pupil method and the pupil shape was approximated as an ellipse. The device mapped the positions of both pupils to the gaze angle and analyzed eye movements at a sampling rate of 500 Hz.

Saccades were elicited by a white circular visual target that jumped horizontally or vertically on a 59.6 × 33.6 cm monitor placed 60 cm in front of the participant. Before performing the task, visually-guided saccades to points 7.5 degrees away in the horizontal and vertical directions were elicited to perform calibration. Since downward saccades, in particular, may interfere with pupil detection and ellipse approximation due to the upper eyelid overlapping the pupil, we visually confirmed that pupil detection was being performed correctly during the calibration phase. For horizontal saccade measurements, the target jumped 5 °, 10 °, 15 °, or 20 °to the right or left at 2–3 s intervals, repeating this pattern 32 times in a pseudo-random unpredictable manner, thereby eliciting 32 visually guided saccades. Of these saccades, those that started and ended at the primary position (straight-ahead position) were extracted, excluding jumps between intermediate positions. A total of 16 jumps (8 centrifugal and 8 centripetal) per measurement were included in the analysis of the horizontal saccades. For vertical saccade measurements, the target jumped 5 °, 10 °, or 15 °upward or downward at 2–3 s intervals, repeating 36 times in the same manner as the horizontal saccade task. Vertical data were extracted as with the horizontal data, and 18 jumps (9 centrifugal and 9 centripetal jumps) per measurement were included in the final analysis.

### 2.3 Eye movement analyses

In this study, we analyzed the maximum velocity, latency, and amplitude of the saccades. For the device used, the start of a saccade was defined as the point at which the velocity exceeded 5 °/s in the direction of the visual targets, and the endpoint was defined as the point at which the velocity decreased below 5 °/s. The maximum velocity of the saccade was the highest velocity observed during the saccade, latency was the time difference between the target jump and the onset of the saccade, and amplitude was the angular displacement of the eye movement. We excluded saccades where the saccade velocity curve sequence was missing or interrupted due to insufficient pupillary tracking, such as during blinking, as they did not allow for the proper evaluation of saccades. We also excluded saccades that occurred before the target moved or within 80 ms after the target began moving, classifying them as anticipatory saccades. Furthermore, to exclude compensatory saccades, we analyzed only the first saccade following target presentation and excluded all subsequent saccades from the analysis.

### 2.4 Statistical analyses

Maximum velocity, latency, and amplitude of the saccades were compared using a paired t-test corrected using the Holm–Bonferroni method. The tests were grouped into 4 families based on directions (centrifugal/centripetal or abduction/adduction for horizontal saccades and centrifugal/centripetal or upward/downward for vertical saccades) and as a result, we conducted six comparative tests for each amplitude. Statistical significance was set at an adjusted *α* = 0.05. The correlation between saccade parameters and age was examined using Pearson’s correlation analysis. All statistical analyses were performed using R software (version 4.3.2).

## 3 Results

### 3.1 Horizontal saccades

For centrifugal saccades, adductions tended to exhibit higher maximum velocities than abductions, especially for larger amplitudes (≥ 15 °; Figures 1, 2, and Tables 1, 2: see A vs. B). Contrastingly, for centripetal saccades, abductions tended to show higher maximum velocities than adductions, with these differences being statistically significant when the amplitudes were sufficiently large (≥ 15 °; Figures 1, 2, and Tables 1, 2: see C vs. D). Thus, saccades that moved within the medial hemifield (between the primary and adducted positions) were faster than those that moved within the lateral hemifield (between the primary and abducted positions), regardless of the movement direction (Tables 1, 2). When comparing centrifugal and centripetal directions, centripetal saccades exhibited significantly higher maximum velocities than centrifugal saccades in both the lateral and medial hemifields when the amplitudes were sufficiently large (≥ 15 °; Tables 1, 2). In contrast to the maximum velocities, no clear trends were observed in the latency or amplitude for the differences between the adduction-abduction and centrifugal-centripetal directions (Tables 3, 4, Supplementary Tables S1, S2, and Supplementary Figures S1, S2).

**Figure 1:**
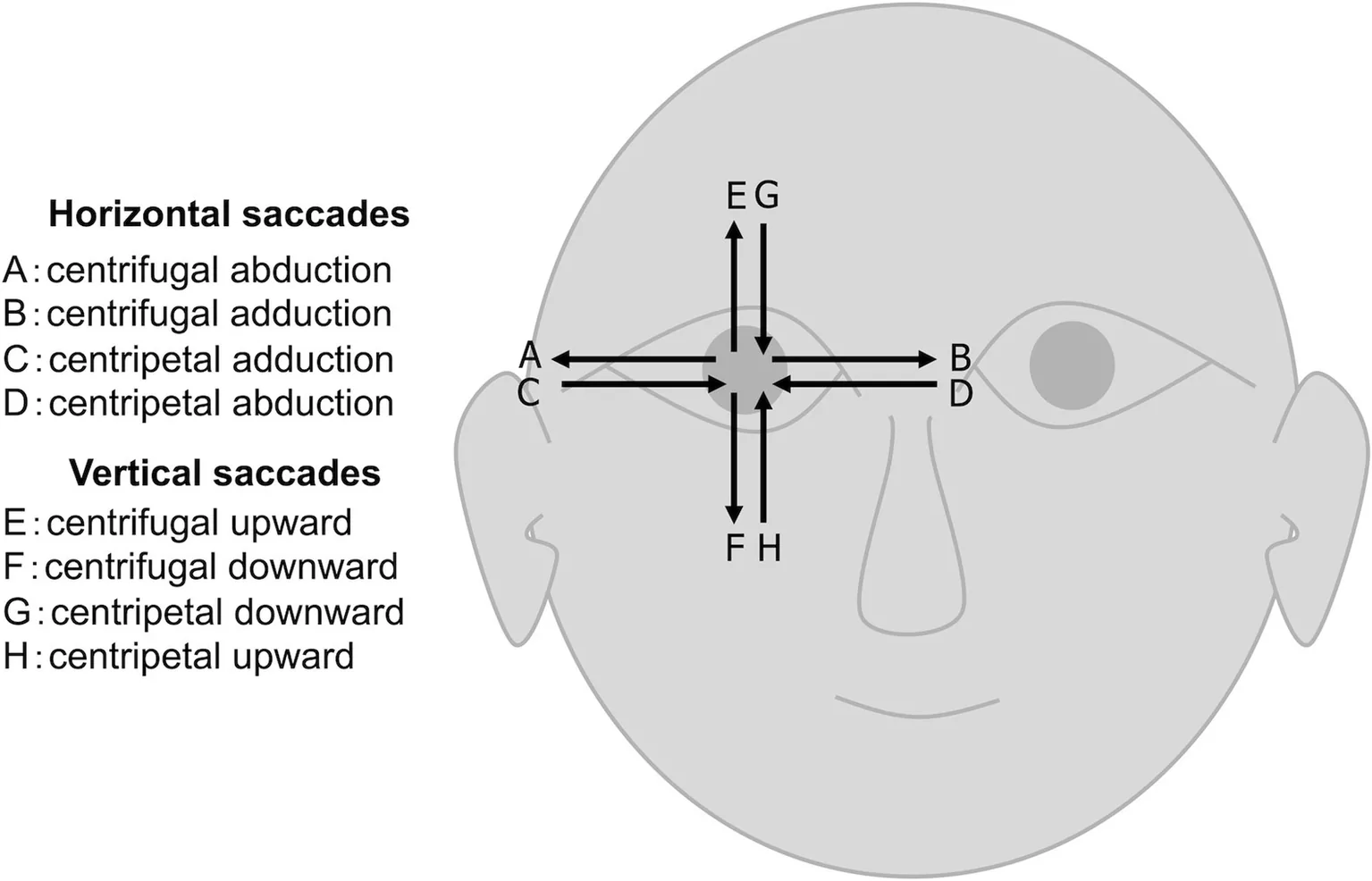
Schematic representation of the 8 saccade directions. Horizontal (saccades A–D) and vertical (saccades E–H) saccades are shown.

**Figure 2:**
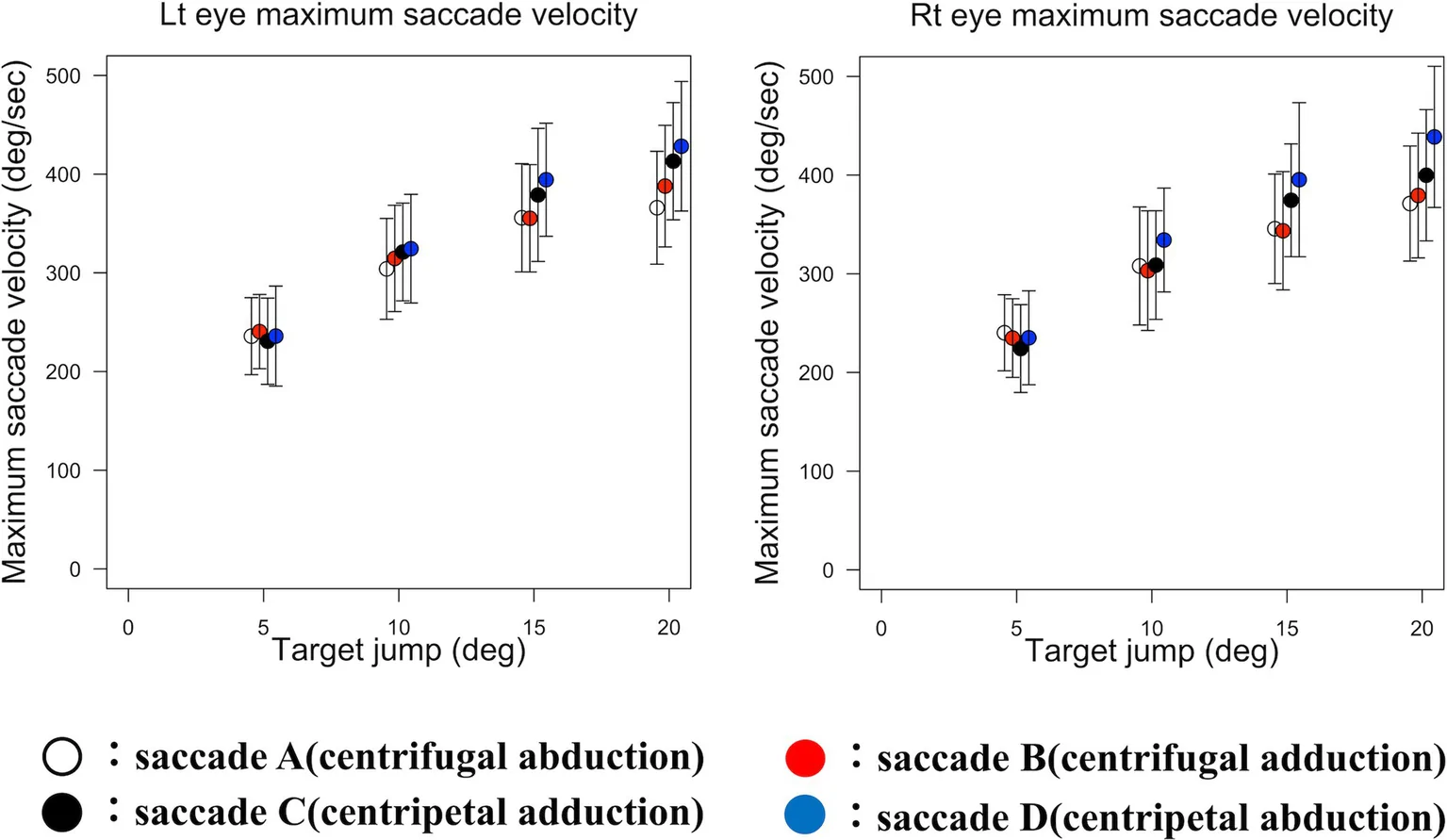
Change in maximum saccade velocity for each horizontal direction. The graphs display the maximum saccade velocity in each direction for amplitudes in 5° increments up to 20°. Red circles represent saccade A, black circles represent saccade B, white circles represent saccade C, and blue circles represent saccade D. See Figure 1 for saccade A–D. Vertical bars on the circles indicate the mean ± SD.

**Table 1:** Maximum velocities of horizontal saccades in 4 directions.

Maximum velocity (deg/s)		A	B	C	D
Left eye	20 degrees	365.99 (57.21)	387.90 (61.58)	413.15 (59.45)	428.28 (65.64)
	15 degrees	335.79 (54.87)	355.25 (54.42)	378.94 (67.39)	394.36 (57.34)
	10 degrees	304.00 (51.08)	314.61 (53.83)	321.14 (49.54)	324.46 (55.01)
	5 degrees	235.85 (39.03)	240.48 (37.61)	230.72 (43.64)	235.92 (50.70)
Right eye	20 degrees	371.17 (58.35)	379.35 (63.18)	399.84 (66.49)	438.71 (71.55)
	15 degrees	345.64 (55.50)	343.57 (59.90)	374.47 (57.18)	395.35 (78.11)
	10 degrees	307.91 (59.84)	303.16 (60.61)	308.83 (55.00)	334.21 (52.63)
	5 degrees	240.21 (38.50)	234.85 (39.84)	224.24 (44.54)	235.15 (47.61)

The letters A–D correspond to the directions A–D in Figure 1.

Data are shown as the mean (SD).

**Table 2:** *P*-values and effect sizes for comparison of maximum velocity of horizontal saccades.

Eye	Comparison	Mean difference $\left(\bar{d}\right)$	95% CI	Effect size (*d_z_*)	Adjusted *p-*value
5 degrees					
Lt eye	A vs. B (B-A)	4.62	[−5.18, 14.42]	0.14	1
	A vs. C (C-A)	−5.13	[−16.61, 6.34]	−0.13	1
	A vs. D (D-A)	0.06	[−12.44, 12.56]	<0.01	0.99
	B vs. C (C-B)	−9.75	[−20.21, 0.70]	−0.27	0.40
	B vs. D (D-B)	−4.56	[−16.96, 7.85]	−0.11	1
	C vs. D (D-C)	5.20	[−10.42, 20.81]	0.10	1
Rt eye	A vs. B (B-A)	−5.35	[−14.61, 3.91]	−0.17	0.75
	A vs. C (C-A)	−15.96	[−26.47, −5.45]	−0.44	0.02*
	A vs. D (D-A)	−5.05	[−15.14, 5.04]	−0.15	0.64
	B vs. C (C-B)	−10.61	[−21.23, 0.01]	−0.29	0.25
	B vs. D (D-B)	0.30	[−11.50, 12.10]	0.01	0.96
	C vs. D (D-C)	10.91	[−3.87, 25.69]	0.21	0.58
10 degrees					
Lt eye	A vs. B (B-A)	10.60	[−1.81, 23.01]	0.25	0.37
	A vs. C (C-A)	17.13	[3.88, 30.39]	0.38	0.06
	A vs. D (D-A)	20.35	[10.43, 30.26]	0.60	0.01*
	B vs. C (C-B)	6.53	[−6.15, 19.21]	0.15	0.61
	B vs. D (D-B)	9.74	[−1.80, 21.29]	0.24	0.28
	C vs. D (D-C)	3.21	[−11.61, 18.04]	0.06	0.65
Rt eye	A vs. B (B-A)	−4.75	[−21.77, 12.27]	−0.08	1
	A vs. C (C-A)	0.93	[−11.32, 13.17]	0.02	0.88
	A vs. D (D-A)	26.30	[15.28, 37.31]	0.69	<0.01*
	B vs. C (C-B)	5.68	[−6.95, 18.31]	0.13	1
	B vs. D (D-B)	31.05	[14.89, 47.21]	0.56	<0.01*
	C vs. D (D-C)	25.37	[12.30, 38.45]	0.56	<0.01*
15 degrees					
Lt eye	A vs. B (B-A)	21.57	[8.81, 34.32]	0.49	<0.01*
	A vs. C (C-A)	45.25	[30.75, 59.75]	0.91	<0.01*
	A vs. D (D-A)	60.67	[48.29, 73.04]	1.42	<0.01*
	B vs. C (C-B)	23.69	[12.61, 34.76]	0.62	<0.01*
	B vs. D (D-B)	39.10	[27.23, 50.98]	0.96	<0.01*
	C vs. D (D-C)	15.42	[1.78, 29.06]	0.33	0.03*
Rt eye	A vs. B (B-A)	−2.07	[−15.89, 11.76]	−0.04	0.77
	A vs. C (C-A)	27.58	[15.76, 39.41]	0.68	<0.01*
	A vs. D (D-A)	50.10	[37.96, 62.25]	1.20	<0.01*
	B vs. C (C-B)	29.65	[15.80, 43.49]	0.62	<0.01*
	B vs. D (D-B)	52.17	[36.27, 68.06]	0.95	<0.01*
	C vs. D (D-C)	22.52	[7.57, 37.47]	0.44	0.01*
20 degrees					
Lt eye	A vs. B (B-A)	22.14	[10.03, 34.26]	0.53	<0.01*
	A vs. C (C-A)	47.39	[35.32, 59.46]	1.14	<0.01*
	A vs. D (D-A)	62.53	[53.69, 71.37]	2.05	<0.01*
	B vs. C (C-B)	25.25	[16.26, 34.24]	0.82	<0.01*
	B vs. D (D-B)	40.38	[26.79, 53.98]	0.86	<0.01*
	C vs. D (D-C)	15.13	[3.88, 26.39]	0.39	<0.01*
Rt eye	A vs. B (B-A)	9.78	[−1.99, 21.56]	0.24	0.16
	A vs. C (C-A)	30.27	[17.79, 42.75]	0.70	<0.01*
	A vs. D (D-A)	69.15	[57.15, 81.15]	1.67	<0.01*
	B vs. C (C-B)	20.48	[10.06, 30.91]	0.57	<0.01*
	B vs. D (D-B)	59.36	[43.84, 74.89]	1.11	<0.01*
	C vs. D (D-C)	38.88	[25.13, 52.63]	0.82	<0.01*

The letters A–D correspond to the directions A–D in Figure 1. The *p*-values were adjusted using the Bonferroni–Holm method (1 family = 6 paired test). Effect sizes are reported as Cohen’s *d_z_* for paired samples.

CI, confidence intervals, *adjusted *p*-value < 0.05.

**Table 3:** Latencies of horizontal saccades in 4 directions.

Saccade latency (s)		A	B	C	D
Left eye	20 degrees	0.179 (0.028)	0.199 (0.031)	0.175 (0.026)	0.171 (0.030)
	15 degrees	0.170 (0.026)	0.184 (0.025)	0.175 (0.027)	0.175 (0.027)
	10 degrees	0.164 (0.033)	0.164 (0.035)	0.166 (0.036)	0.154 (0.029)
	5 degrees	0.169 (0.034)	0.177 (0.043)	0.157 (0.039)	0.158 (0.031)
Right eye	20 degrees	0.198 (0.031)	0.180 (0.028)	0.171 (0.030)	0.174 (0.026)
	15 degrees	0.183 (0.025)	0.171 (0.027)	0.177 (0.027)	0.174 (0.027)
	10 degrees	0.163 (0.035)	0.165 (0.032)	0.155 (0.029)	0.165 (0.036)
	5 degrees	0.176 (0.043)	0.170 (0.034)	0.159 (0.031)	0.157 (0.039)

The letters A–D correspond to the directions A–D in Figure 1.

Data are shown as the mean (SD).

**Table 4:** Amplitudes of horizontal saccades in 4 directions.

Saccade gain		A	B	C	D
Left eye	20 degrees	0.879 (0.069)	0.898 (0.072)	0.881 (0.113)	0.884 (0.094)
	15 degrees	0.889 (0.095)	0.923 (0.090)	0.923 (0.118)	0.923 (0.079)
	10 degrees	0.916 (0.110)	0.931 (0.141)	0.942 (0.111)	0.889 (0.105)
	5 degrees	0.931 (0.129)	0.993 (0.106)	0.941 (0.190)	0.921 (0.138)
Right eye	20 degrees	0.866 (0.093)	0.915 (0.065)	0.898 (0.100)	0.860 (0.110)
	15 degrees	0.880 (0.100)	0.922 (0.099)	0.940 (0.081)	0.906 (0.121)
	10 degrees	0.883 (0.135)	0.951 (0.149)	0.904 (0.116)	0.924 (0.163)
	5 degrees	0.942 (0.107)	0.975 (0.131)	0.937 (0.152)	0.906 (0.189)

The amplitude was expressed as gain (eye position)/(target position).

The letters A–D correspond to the directions A–D in Figure 1.

Data are shown as the mean (SD).

### 3.2 Vertical saccades

In the centrifugal direction, upward saccades tended to exhibit higher maximum velocities than downward saccades, with this difference reaching statistical significance at a 15 ° amplitude (Figures 1, 3 and Tables 5, 6; see E vs. F). Contrastingly, in the centripetal direction, downward saccades tended to show higher maximum velocities than upward saccades, with a statistically significant difference observed for the 15 ° amplitude (Tables 5, 6; see G vs. H). When comparing the centrifugal and centripetal directions of saccades that moved within the upper hemifield (between the primary and upward positions), centripetal saccades (downward saccades in the upper hemifield) showed a tendency for higher maximum velocities than centrifugal saccades (upward saccades in the upper hemifield) at a 15 ° amplitude (Tables 5, 6). When comparing the centrifugal and centripetal directions for saccades that moved within the lower hemifield (between the primary and downward positions), centrifugal saccades (downward saccades in the lower hemifield) were slightly faster than centripetal saccades (upward saccades in the lower hemifield). However, there was no statistically significant difference; they were almost identical (Tables 5, 6). Overall, the vertical saccades at a 15 ° amplitude showed a tendency to be faster in the following order: centripetal direction in the upper hemifield, centrifugal direction in the upper hemifield, centrifugal direction in the lower hemifield, and centripetal direction in the lower hemifield.

**Figure 3:**
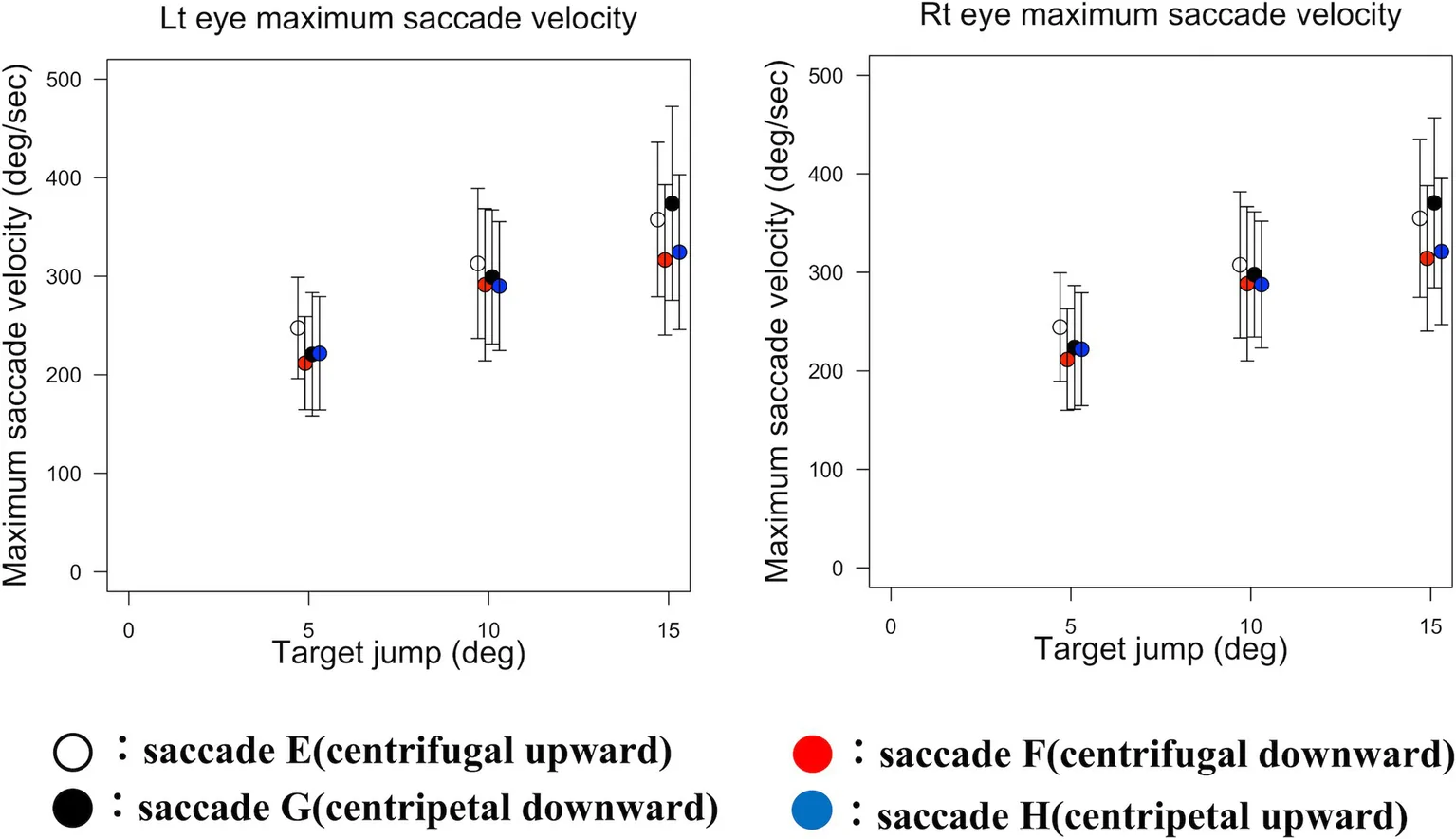
Change in maximum saccade velocity for each vertical direction. The graphs display the maximum saccade velocity in each direction for amplitudes in 5° increments up to 15°. Red circles represent saccades E, black circles represent saccades F, white circles represent saccades G, and blue circles represent saccade H. See Figure 1 for saccades E–H. Vertical bars on the circles indicate the mean ± SD.

**Table 5:** Maximum velocities of vertical saccades in 4 directions.

Maximum velocity (deg/s)		E	F	G	H
Left eye	15 degrees	357.57 (78.42)	316.60 (76.33)	373.89 (98.40)	324.46 (78.58)
	10 degrees	312.99 (76.21)	291.38 (77.21)	299.23 (68.05)	290.03 (65.36)
	5 degrees	247.56 (51.44)	211.78 (47.28)	220.78 (62.57)	221.80 (57.51)
Right eye	15 degrees	354.90 (80.26)	314.22 (73.86)	370.59 (86.19)	321.12 (74.09)
	10 degrees	307.59 (74.23)	288.43 (78.23)	297.88 (63.48)	287.62 (64.36)
	5 degrees	244.44 (55.05)	211.49 (51.57)	223.85 (62.85)	221.95 (57.28)

The letters E–H correspond to the E–H direction in Figure 1.

Data are shown as the mean (SD).

**Table 6:** *P*-values and effect sizes for comparison of maximum velocity of vertical saccades.

Eye	Comparison	Mean difference $\left(\bar{d}\right)$	95% CI	Effect size (*d_z_*)	Adjusted *p-*value
5 degrees					
Lt eye	E vs. F (F-E)	−35.78	[−52.21, −19.35]	−0.63	<0.01*
	E vs. G (G-E)	−26.79	[−40.75, −12.82]	−0.56	<0.01*
	E vs. H (H-E)	−25.76	[−39.37, −12.16]	−0.55	<0.01*
	F vs. G (G-F)	8.99	[−5.85, 23.83]	0.18	0.69
	H vs. F (H-F)	10.02	[−9.44, 29.48]	0.15	0.61
	G vs. H (H-G)	1.02	[−20.92, 22.97]	0.01	0.93
Rt eye	E vs. F (F-E)	−32.95	[−52.70, −13.20]	−0.48	<0.01*
	E vs. G (G-E)	−20.59	[−36.81, −4.37]	−0.37	0.06
	E vs. H (H-E)	−22.49	[−35.02, −9.96]	−0.52	<0.01*
	F vs. G (G-F)	12.36	[−7.10, 31.81]	0.18	0.62
	F vs. H (H-F)	10.46	[−7.79, 28.71]	0.17	0.51
	G vs. H (H-G)	−1.90	[−23.47, 19.67]	−0.03	0.86
10 degrees					
Lt eye	E vs. F (F-E)	−21.61	[−45.69, 2.47]	−0.26	0.39
	E vs. G (G-E)	−13.76	[−34.15, 6.64]	−0.20	0.73
	E vs. H (H-E)	−22.96	[−34.88, −11.03]	−0.56	<0.01*
	F vs. G (G-F)	7.85	[−8.40, 24.10]	0.14	0.67
	H vs. F (H-F)	−1.35	[−22.66, 19.96]	−0.02	0.90
	G vs. H (H-G)	−9.20	[−26.14, 7.74]	−0.16	0.84
Rt eye	E vs. F (F-E)	−19.16	[−40.85, 2.53]	−0.26	0.41
	E vs. G (G-E)	−9.71	[−29.18, 9.75]	−0.14	0.64
	E vs. H (H-E)	−19.97	[−32.97, −6.96]	−0.45	0.02*
	F vs. G (G-F)	9.45	[−3.67, 22.57]	0.21	0.62
	F vs. H (H-F)	−0.80	[−20.29, 18.69]	−0.01	0.93
	G vs. H (H-G)	−10.25	[−26.19, 5.68]	−0.19	0.61
15 degrees					
Lt eye	E vs. F (F-E)	−40.97	[−64.30, −17.64]	−0.51	<0.01*
	E vs. G (G-E)	16.32	[−8.05, 40.68]	0.19	0.37
	E vs. H (H-E)	−33.12	[−50.50, −15.73]	−0.55	<0.01*
	F vs. G (G-F)	57.28	[35.02, 79.54]	0.75	<0.01*
	H vs. F (H-F)	7.85	[−14.60, 30.30]	0.10	0.49
	G vs. H (H-G)	−49.43	[−75.23, −23.63]	−0.56	<0.01*
Rt eye	E vs. F (F-E)	−40.68	[−64.55, −16.82]	−0.49	<0.01*
	E vs. G (G-E)	15.70	[−8.00, 39.39]	0.19	0.38
	E vs. H (H-E)	−33.77	[−48.78, −18.77]	−0.65	<0.01*
	F vs. G (G-F)	56.38	[37.46 75.30]	0.87	<0.01*
	F vs. H (H-F)	6.91	[−15.95, 29.76]	0.09	0.55
	G vs. H (H-G)	−49.47	[−73.45, −25.49]	−0.60	<0.01*

The letters E–H correspond to the E–H direction in Figure 1. The *p*-values were adjusted using the Bonferroni–Holm method (1 family = 6 paired test). Effect sizes are reported as Cohen’s *d_z_* for paired samples.

CI, confidence intervals, *adjusted *p*-value < 0.05.

Regarding saccade latencies at a 15 ° amplitude, centripetal saccades generally exhibited shorter latencies than centrifugal saccades in both the upward and downward directions, with the shortest latencies observed for centripetal downward saccades (Table 7, Supplementary Table S3, and Supplementary Figure S3).

**Table 7:** Latencies of vertical saccades in 4 directions.

Saccade latency (s)		E	F	G	H
Left eye	15 degrees	0.194 (0.029)	0.196 (0.031)	0.171 (0.031)	0.185 (0.026)
	10 degrees	0.194 (0.028)	0.177 (0.021)	0.168 (0.025)	0.179 (0.024)
	5 degrees	0.221 (0.059)	0.181 (0.046)	0.173 (0.050)	0.198 (0.033)
Right eye	15 degrees	0.194 (0.028)	0.196 (0.032)	0.169 (0.031)	0.184 (0.029)
	10 degrees	0.194 (0.029)	0.177 (0.021)	0.167 (0.026)	0.179 (0.025)
	5 degrees	0.221 (0.059)	0.181 (0.045)	0.173 (0.049)	0.198 (0.032)

The letters E–H correspond to the E–H direction in Figure 1.

Data are shown as the mean (SD).

Regarding saccade amplitudes evaluated based on gain (eye position/target position), upward saccades tended to show larger amplitudes than downward saccades in both the centrifugal and centripetal directions, with statistically significant differences observed in the centrifugal direction (Table 8, Supplementary Table S4, and Supplementary Figure S4). When comparing centrifugal and centripetal saccades within the upper hemifield, upward (centrifugal) saccades had significantly larger amplitudes. Similarly, in the lower hemifield, upward (centripetal) saccades also had significantly larger amplitudes at amplitudes ≥ 10 °. Overall, the vertical saccades tended to exhibit larger amplitudes in the following order: upward (centrifugal) in the upper hemifield, upward (centripetal) in the lower hemifield, and downward in both the upper (centripetal) and lower (centrifugal) direction hemifields (Table 8, Supplementary Table S4, and Supplementary Figure S4).

**Table 8:** Amplitudes of vertical saccades in 4 directions.

Saccade gain		E	F	G	H
Left eye	15 degrees	0.982 (0.112)	0.812 (0.119)	0.888 (0.187)	0.942 (0.181)
	10 degrees	1.033 (0.127)	0.859 (0.132)	0.869 (0.152)	0.992 (0.168)
	5 degrees	1.137 (0.151)	0.901 (0.153)	0.916 (0.210)	0.963 (0.202)
Right eye	15 degrees	0.976 (0.113)	0.816 (0.111)	0.887 (0.181)	0.939 (0.188)
	10 degrees	1.039 (0.148)	0.865 (0.135)	0.851 (0.145)	0.997 (0.158)
	5 degrees	1.113 (0.162)	0.911 (0.151)	0.919 (0.206)	0.951 (0.196)

The amplitude was expressed as gain (eye position)/(target position).

The letters E–H correspond to the E–H direction in Figure 1.

Data are shown as the mean (SD).

### 3.3 Effects of age on saccades

In this study, no significant correlation was observed between saccade velocity and age across all directions and amplitudes. Regarding saccade amplitude, a significant negative correlation with age was found only for right-eye abduction and left-eye adduction during a 10 ° centrifugal rightward saccade, for right-eye adduction and left-eye abduction during a 15 ° centripetal leftward saccade, and for left-eye depression during a 10 ° centripetal downward saccade. No significant correlation with age was observed for the other saccades. Regarding saccadic latency, a significant positive correlation with age was observed only for left-eye elevation during 5 ° upward centrifugal saccades and for right- and left-eye elevation during 5 ° upward centripetal saccades. No significant correlation with age was found for the other saccades (Supplementary Tables S5, S6; Supplementary Figures S5–S16).

## 4 Discussion

In this study, we measured centrifugal, centripetal, horizontal, and vertical saccades under the same conditions and revealed both adduction-abduction and centrifugal-centripetal asymmetries for each type of saccade.

### 4.1 Horizontal saccade

Our study showed that, in the centrifugal direction (starting from the primary position), adduction saccades were faster than abduction saccades when amplitudes were sufficiently large (≥ 15 °). The faster adduction in centrifugal horizontal saccades was consistent with the results of our previous study, in which we compared centrifugal adduction and abduction saccades at a 15 ° amplitude (4). However, other reports have shown that abduction saccades are faster when smaller-amplitude saccades (<15 °) (7, 8). Indeed, even in our study, small-amplitude saccades did not show significantly faster adduction. The velocity advantage of adduction in centrifugal horizontal saccades appears to become more pronounced with larger-amplitude saccades. Conversely, for centripetal horizontal saccades, abduction saccades were faster than adduction saccades when the amplitudes were sufficiently large. Thus, in the horizontal direction, saccades within the medial hemifield were faster than those within the lateral hemifield, regardless of movement direction. In the centrifugal direction, the saccade-vergence interaction is speculated to contribute to the adduction-abduction asymmetry (10, 11). Vergence is a disconjugate eye movement that primarily occurs within the medial hemifield and involves both the centrifugal (convergence) and centripetal (divergence) directions. Although the velocity of divergence is typically reported to be lower than that of convergence (12, 13), it has been hypothesized that this additional divergence force could increase the abduction velocity within the medial hemifield, as the convergence force enhances the adduction velocity. However, no clear evidence of disconjugate eye movements or a convergence component was observed in the saccades examined in this study. Thus, our findings did not provide support for this hypothesis. To further test this hypothesis, experiments under different conditions, such as varying the target distance, will likely be necessary. Additionally, there is also the possibility that vergence oscillations occur during conjugate saccades, probably arising centrally from an integrative binocular controller (14) and differences in the adduction and abduction directions of the elastic tension of intraorbital tissues, including the extraocular muscles (15) are involved.

Our study not only showed adduction-abduction asymmetries, but also centrifugal-centripetal asymmetries in horizontal saccades; centripetal saccades were faster than centrifugal saccades in both the lateral and medial hemifields. Faster centripetal saccades in the horizontal direction have been previously reported (1–3, 5–7). Because centripetal saccades exhibit both faster velocities and shorter latencies, a bias in the central eye movement-generating mechanism has been proposed that helps return the eye to its primary position for a more efficient search (16). However, in our study, shorter latencies were not as prominent as the faster velocities observed in the centripetal saccades. While velocity reflects the motion characteristics of a saccade, depending on the direction, amplitude, and initial eye position, latency primarily depends on saccade initiation by the central nervous system. Therefore, in our study, mechanical resistance, another proposed mechanism for faster centripetal saccades (17), is more likely to explain the faster velocities than the bias in the central eye movement-generating mechanism. Specifically, when the eye is located away from its primary position, it is subjected to a centripetal elastic restoring force.

### 4.2 Vertical saccades

Our study showed that upward saccades tended to be faster than downward saccades in the centrifugal direction, whereas downward saccades tend to be faster than upward saccades in the centripetal direction. In other words, the saccades moving within the upper hemifield were faster than those moving within the lower hemifield, regardless of the centrifugal-centripetal direction. Upward-downward asymmetry has been previously described in several reports; however, the results varied across studies. Some reports indicated a faster saccade velocity in the upward direction (18, 19), whereas others showed greater downward velocities (20–22). Other studies found no significant difference between upward and downward saccade velocities (23–25). Indeed, in our study, the velocity of vertical saccades differed depending on the conditions, that is, whether the saccade was centrifugal or centripetal. Our results suggest that the velocity of vertical saccades is more likely to be influenced by whether the eye is moving in the upper or lower hemifield rather than by the direction of movement (upward or downward). If this is the case, the upper eyelid may slow the saccade velocity in the lower hemifield. The upper eyelid moves in conjunction with the eyeball. Thus, when looking upwards (in the upper hemifield), the palpebral fissures enlarge, whereas when looking downward (in the lower hemifield), they narrow. Narrowing of the palpebral fissure may increase the viscous frictional resistance due to the increased coverage of the eyeball by the eyelids. This additional resistance may explain the slower saccades observed when moving within the lower hemifield (Supplementary File). Since no studies have investigated the characteristics of vertical saccade velocity based on the location of movement (upper versus lower hemifield) rather than the direction of movement, further verification of the velocity advantage of saccades in the upper hemifield, as observed in our study, is necessary. In this study, we confirmed that the pupil was accurately approximated by an ellipse for each subject during the vertical calibration procedure, including during downgaze. Consistent with this finding, no differences in the amount of missing data or tracking accuracy were observed between upward and downward eye positions. Most of the missing data in this study were attributable to blinking rather than to difficulties in pupil detection caused by eyelid occlusion or other factors. Therefore, it is unlikely that increased eyelid coverage of the pupil during downgaze substantially affected pupil detection or ellipse fitting in the video-based eye-tracking system.

In our study, centripetal vertical saccades may have been faster than centrifugal vertical saccades when moving within the upper hemifield. One possible mechanism for the centripetal velocity advantage is the centripetal elastic restoring force, as suggested for horizontal saccades. However, this centripetal velocity advantage may have been masked by additional resistance from the eyelids when the saccades moved within the lower hemifield.

Additionally, our study found that centripetal saccades tended to have shorter latencies than centrifugal saccades, particularly within the upper hemifield. Latency primarily depends on the initiation of saccades by the central nervous system, rather than on mechanical resistance. Thus, one possible explanation for the shorter latencies in the centripetal vertical saccades may be a bias in the central eye movement-generating mechanism that helps return the eye to the primary position (26–28), or a bias in visual processing that aids in rapidly perceiving the visual target located in the straight-ahead position (16, 29–31). The shorter latencies also raise the possibility that, in addition to the elastic restoring force, biases in the central eye movement generation mechanism and/or visual processing may play a role in the velocity advantage of centripetal upper hemifield vertical saccades.

In our study, the amplitudes of the upward saccades were consistently larger than those of the downward saccades. However, contrary to our results, other studies reported larger downward saccade amplitudes (20, 22). This discrepancy may be due to differences in the experimental conditions. The mechanical effects of the eyelids or an interaction between the superior levator and supraduction muscles (32) may be involved in the upward-downward amplitude asymmetry, although the precise mechanism remains unknown.

This study had several limitations, including a small sample size and limited variety of saccade tasks. Saccades were only measured up to ±20 ° horizontally and ±15 ° vertically due to the constraints of the monitor used for target presentation. Another limitation is that, due to the constraints of the equipment used in this study, the number of saccades typically included in each horizontal and vertical amplitude ranged from 1 to 4 per subject, which may have caused slight variations in the saccade measurement results. Although no substantial correlations with age were observed, the potential influence of variability in participant age on the results of this study cannot be completely excluded. Another potential limitation of this study is that the comparison of maximum velocities was based solely on the measured values. The maximum velocity of a saccade is strongly influenced by its amplitude, and differences in amplitude gain may therefore affect saccade velocity. When comparing maximum saccade velocities across directions using “adjusted velocities,” calculated by dividing the measured velocity by the amplitude gain, we observed some differences in the directional effects reported in this study. Specifically, for large-amplitude vertical centrifugal saccades (i.e., movements from the center toward the periphery), adjusted velocities were higher in the downward direction (Supplementary Tables S7–S10). However, because the programmed saccade amplitude for a given target should be identical, the observed variability in measured amplitude, like that in velocity, may also be influenced by the mechanical resistance of the periocular tissues. Therefore, the interpretation of these adjusted values remains challenging. It may be beneficial for future research to measure the mechanical resistance to eye movements using methods such as the forced duction test.

## 5 Conclusion

Saccades are generally thought to be generated by a uniform mechanism that depends on the distance from a visual target. However, significant adduction-abduction and centrifugal-centripetal asymmetries were observed in both the horizontal and vertical saccades in this study. These asymmetries may be strongly related to the mechanical resistance in periocular tissues.

## Data Availability

The raw data supporting the conclusions of this article will be made available by the authors, without undue reservation.
