# Supplementary material for: Directional differences in saccades
Source: Front Neurol. 2026 Oct 23;17:1945647. doi: 10.3389/fneur.2026.1945647 (PMC13741797; doi:10.3389/fneur.2026.1945647)
Supplement: Supplementary file 1 [file Supplementary_file_2.pdf]

Supplementary Figures and Tables

1 Supplementary Figures

Figure S1 The change of saccade latency in each horizontal direction (saccade A-D in Figure 1)

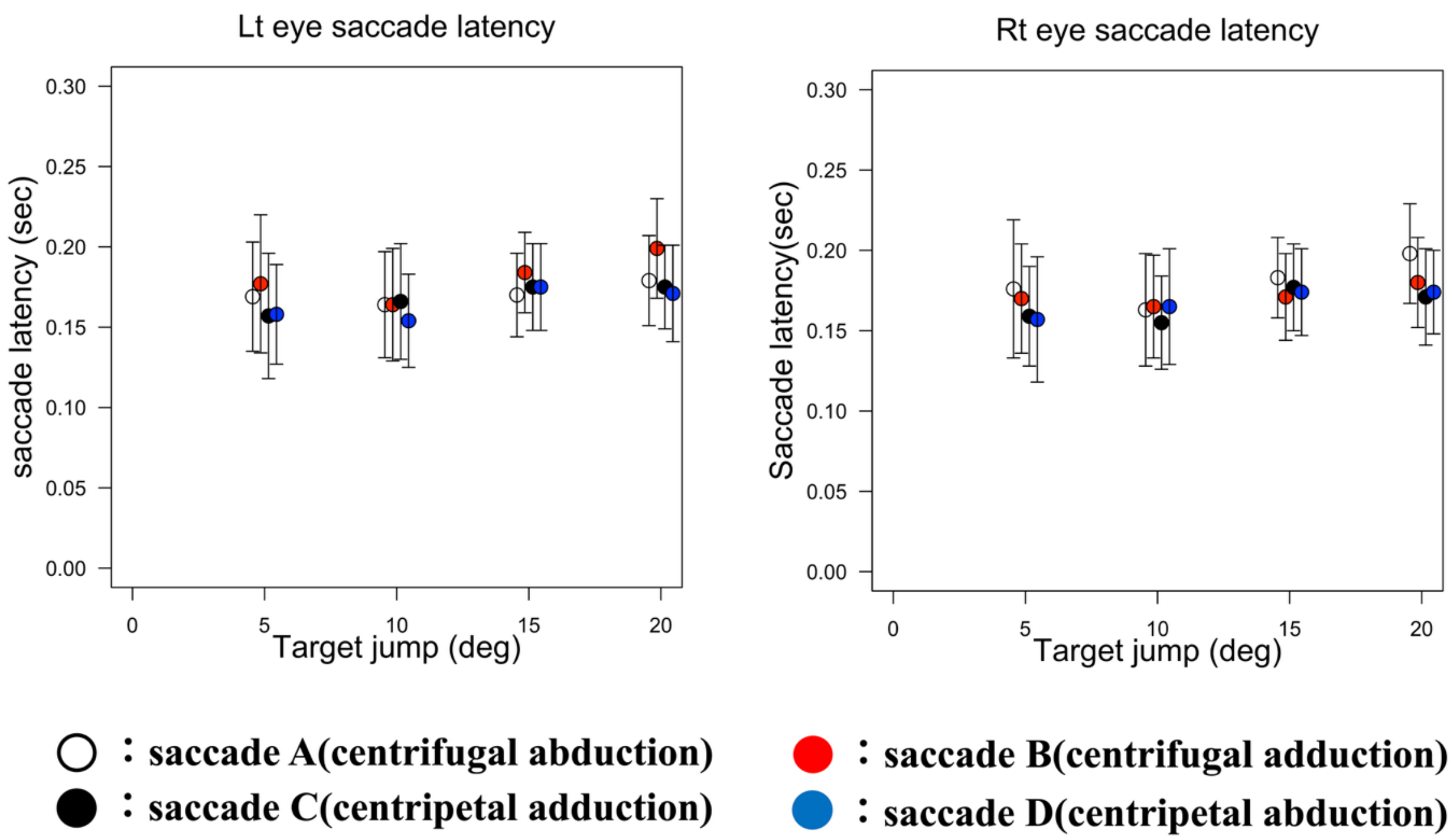

The graphs show the saccade latency in each direction for every 5 °up to 20 °. Red circles present saccade A, black circles present saccade B, white circles present saccade C, blue circle present saccade D. Vertical bars on the circles mean ±SD.

Figure S2 The change of saccade amplitude in each horizontal direction (saccade A-D in Figure 1)

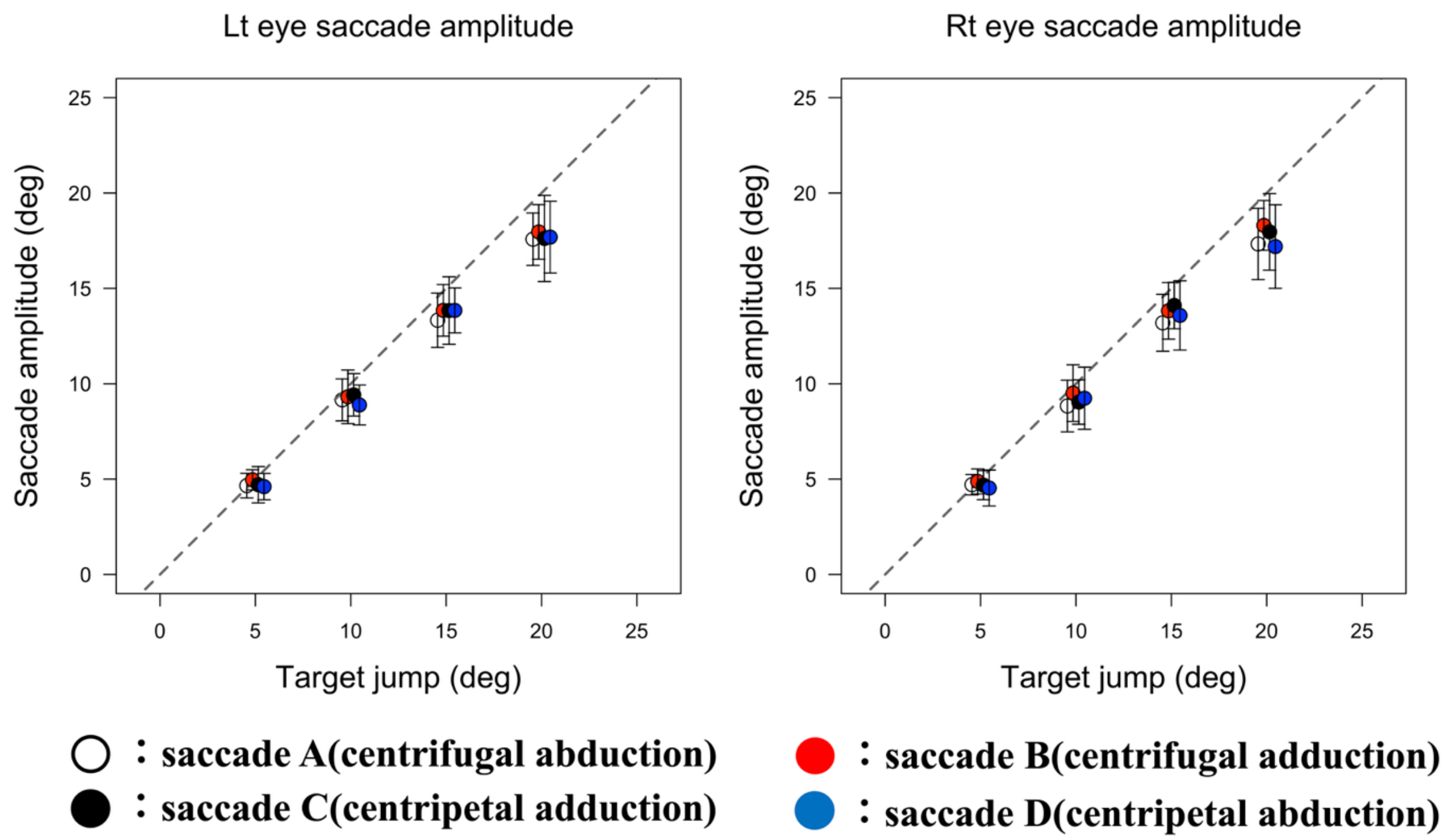

The graphs show the saccade amplitude in relation to the target amplitude every 5 °–20 °. The dashed diagonal indicates the unity gain. Red circles present saccade A, black circles present saccade B, white circles present saccade C, blue circle present saccade D. Vertical bars on the circles mean ±SD.

Figure S3 The change of saccade latency in each vertical direction (saccade E-H in Figure 1)

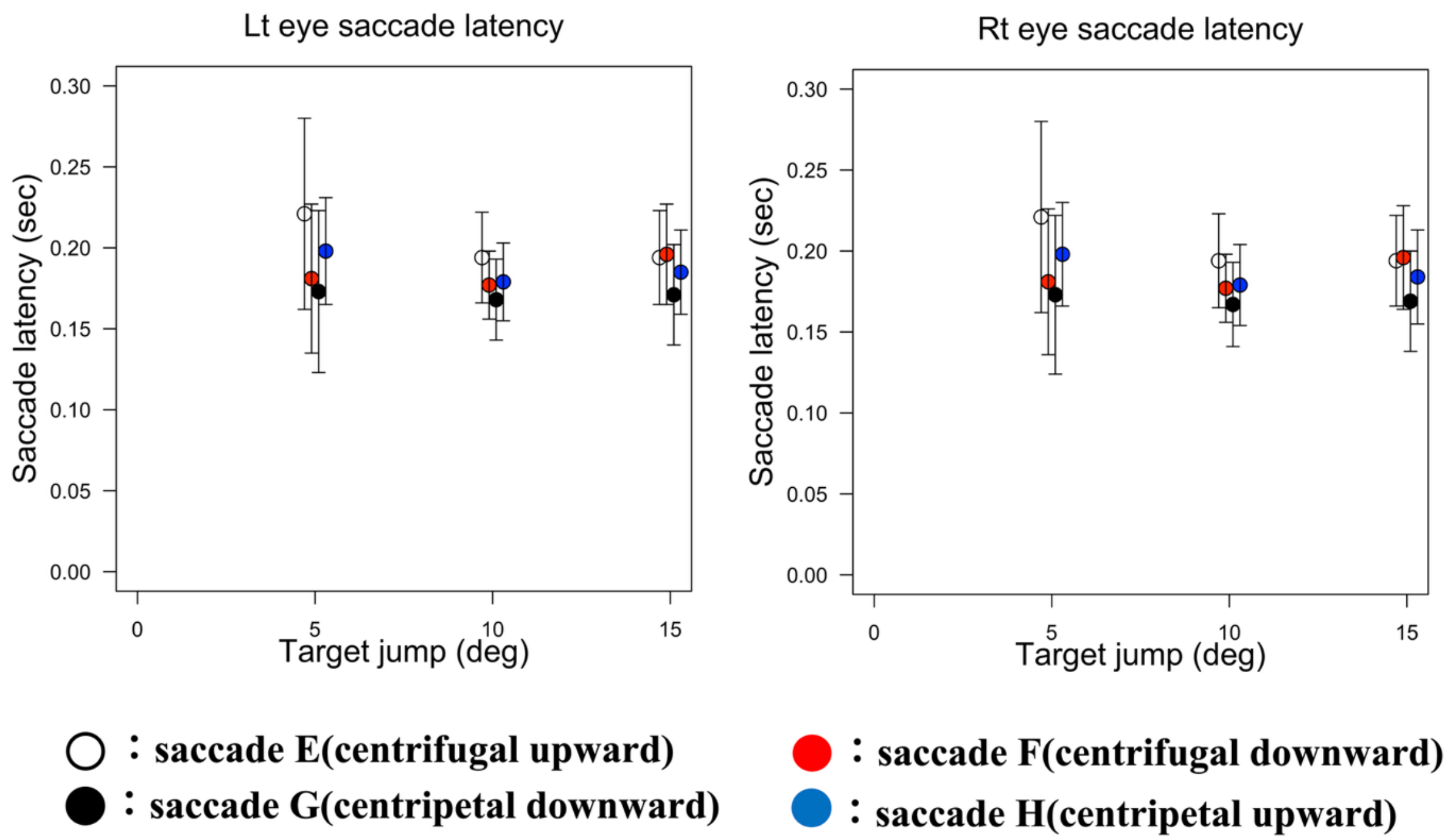

The graphs show the saccade latency in each direction for every 5 °up to 15 °. Red circles present saccade E, black circles present saccade F, white circles present saccade G, blue circle present saccade H. Vertical bars on the circles mean ±SD.

Figure S4 The change of saccade amplitude in each vertical direction (saccade E-H in Figure 1)

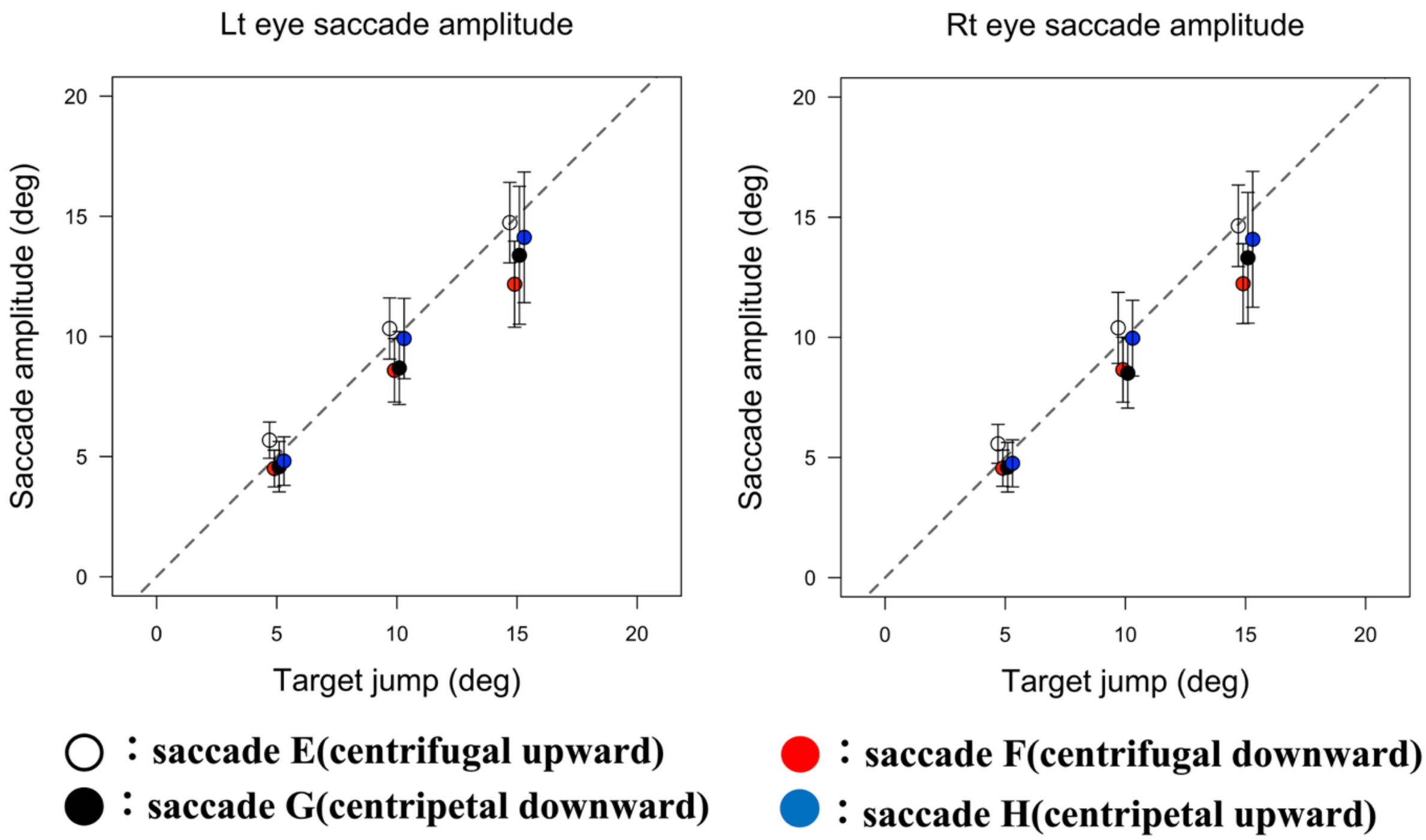

The graphs show the saccade amplitude in relation to the target amplitude for every 5–15 °. The dashed diagonal indicates the unity gain. Red circles present saccade E, black circles present saccade F, white circles present saccade G, blue circle present saccade H. Vertical bars on the circles mean ±SD.

Figure S5 The relationship between age and horizontal centrifugal saccade velocity in each amplitude (saccade A,B in Figure 1)

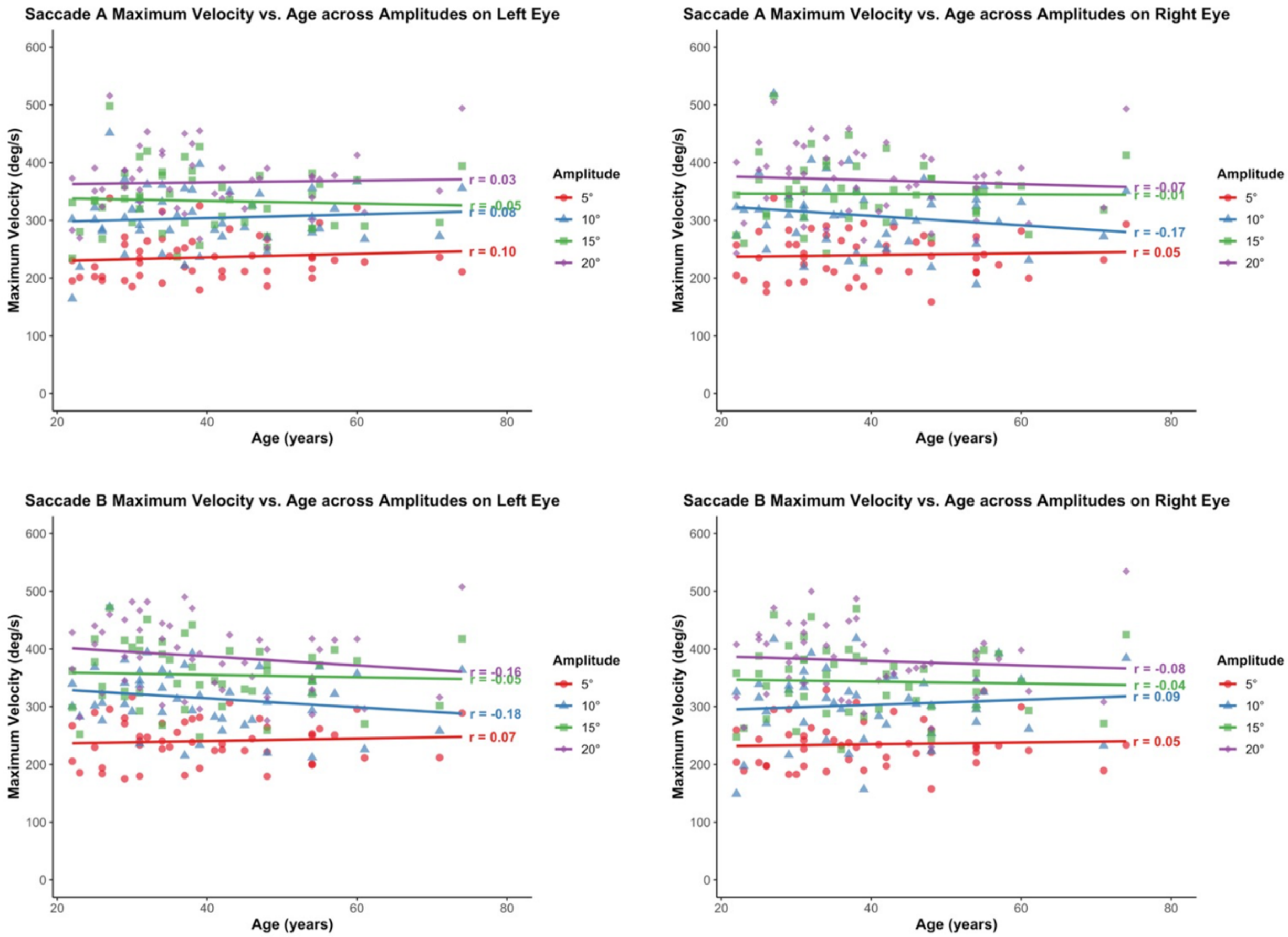

The left column shows data for the left eye, and the right column shows the right eye. Red circles and regression line present 5 °, blue triangles and regression line present saccade 10 °, green squares and regression line present 15 °, purple rhombuses and regression line present 20 °. “r” denotes the correlation coefficient in Pearson's correlation analysis.

Figure S6 The relationship between age and horizontal centripetal saccade velocity in each amplitude (saccade C,D in Figure 1)

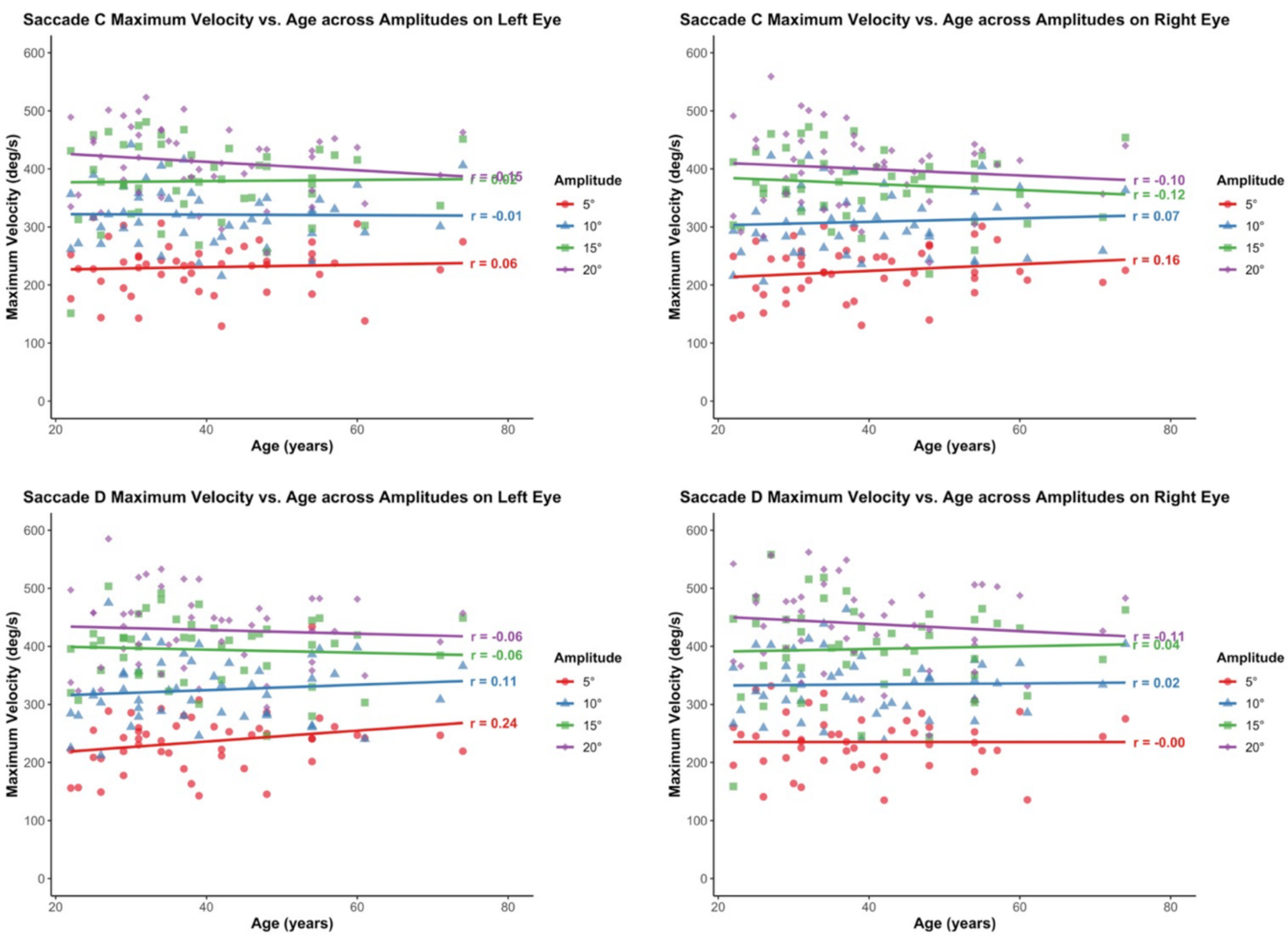

The left column shows data for the left eye, and the right column shows the right eye. Red circles and regression line present 5 °, blue triangles and regression line present saccade 10 °, green squares and regression line present 15 °, purple rhombuses and regression line present 20 °. “r” denotes the correlation coefficient in Pearson's correlation analysis.

Figure S7 The relationship between age and horizontal centrifugal saccade latency in each amplitude (saccade A,B in Figure 1)

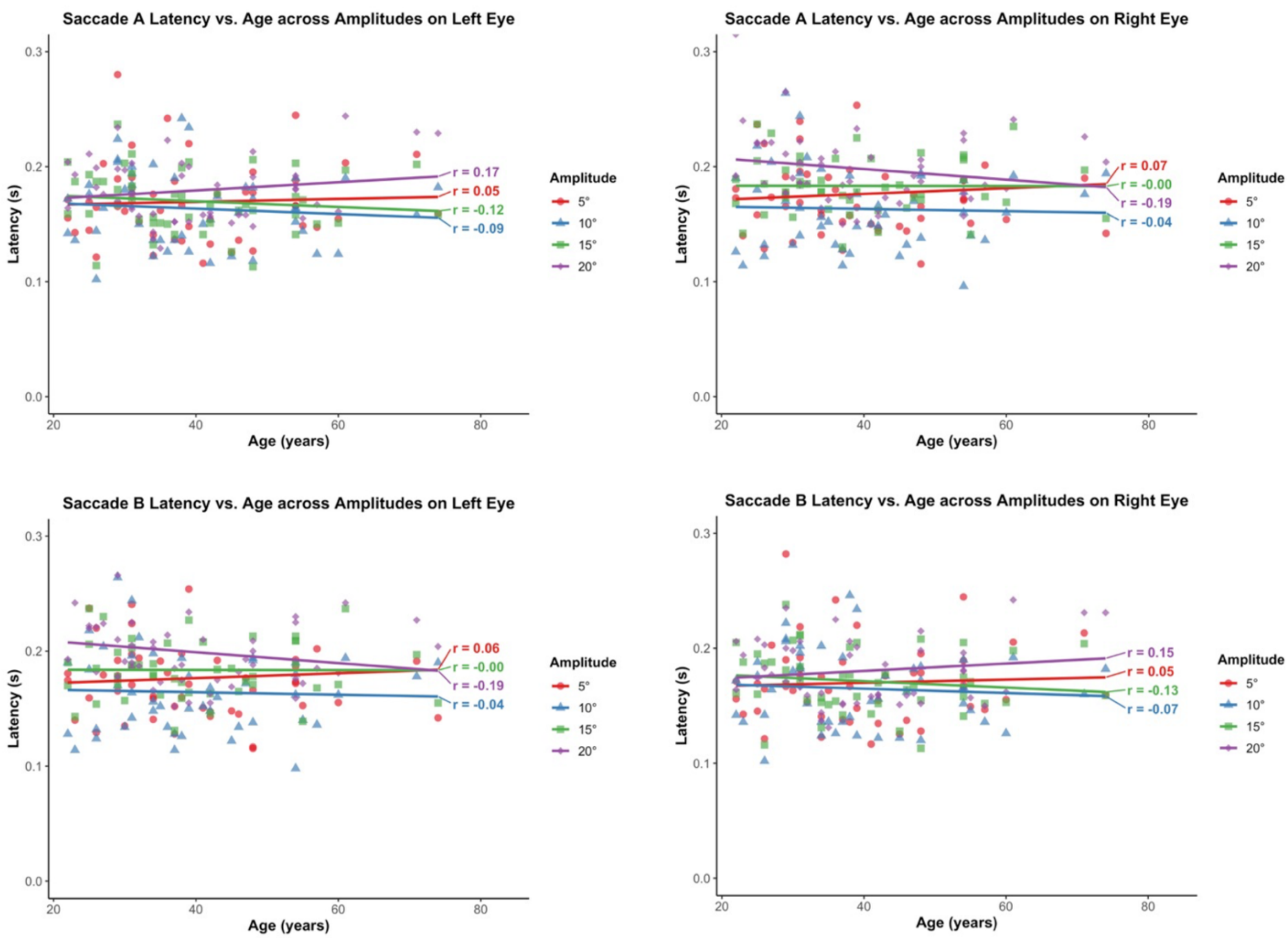

The left column shows data for the left eye, and the right column shows the right eye. Red circles and regression line present 5 °, blue triangles and regression line present saccade 10 °, green squares and regression line present 15 °, purple rhombuses and regression line present 20 °. “r” denotes the correlation coefficient in Pearson's correlation analysis.

Figure S8 The relationship between age and horizontal centripetal saccade latency in each amplitude (saccade C,D in Figure 1)

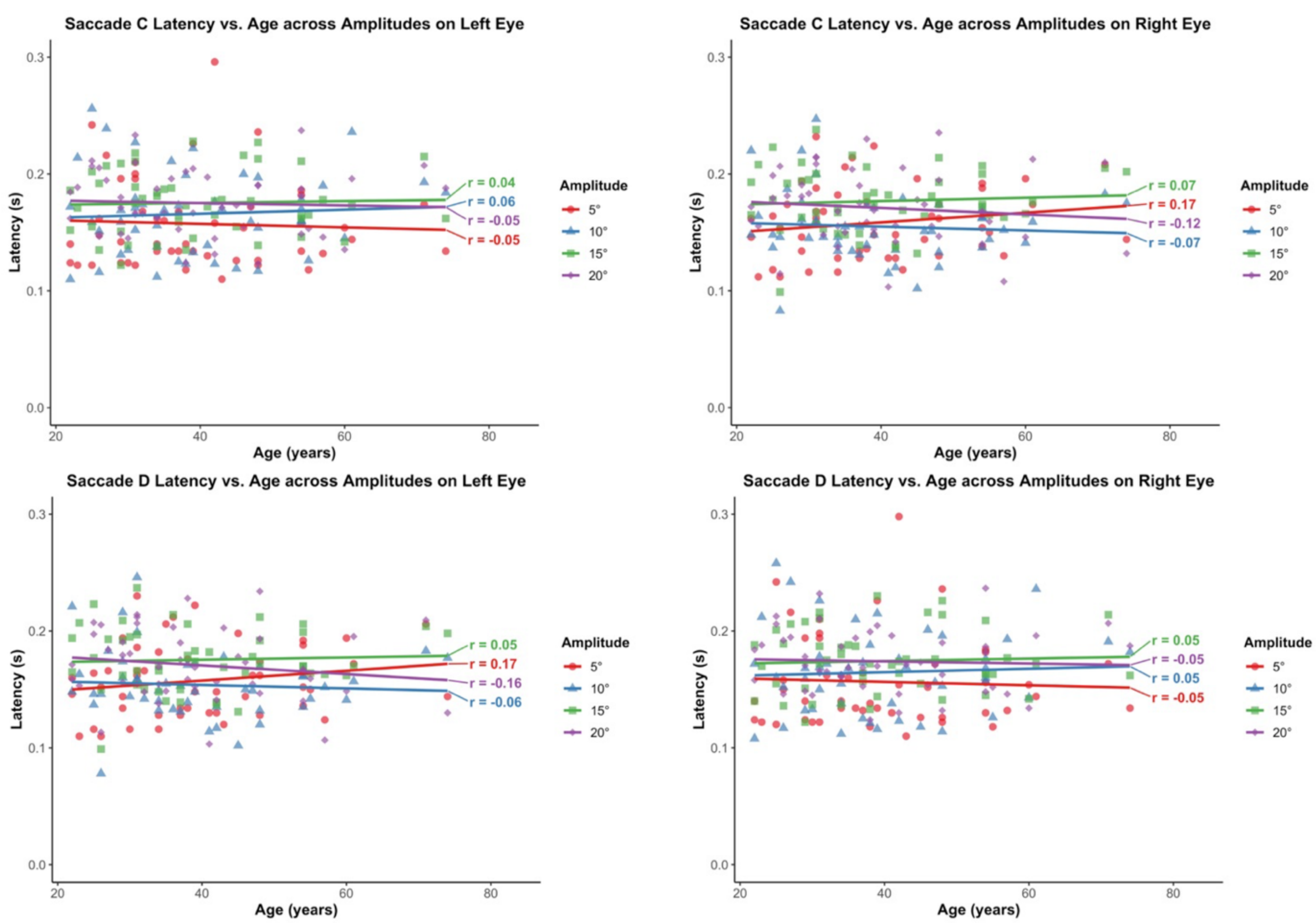

The left column shows data for the left eye, and the right column shows the right eye. Red circles and regression line present 5 °, blue triangles and regression line present saccade 10 °, green squares and regression line present 15 °, purple rhombuses and regression line present 20 °. “r” denotes the correlation coefficient in Pearson's correlation analysis.

Figure S9 The relationship between age and horizontal centrifugal saccade gain in each amplitude (saccade A,B in Figure 1)

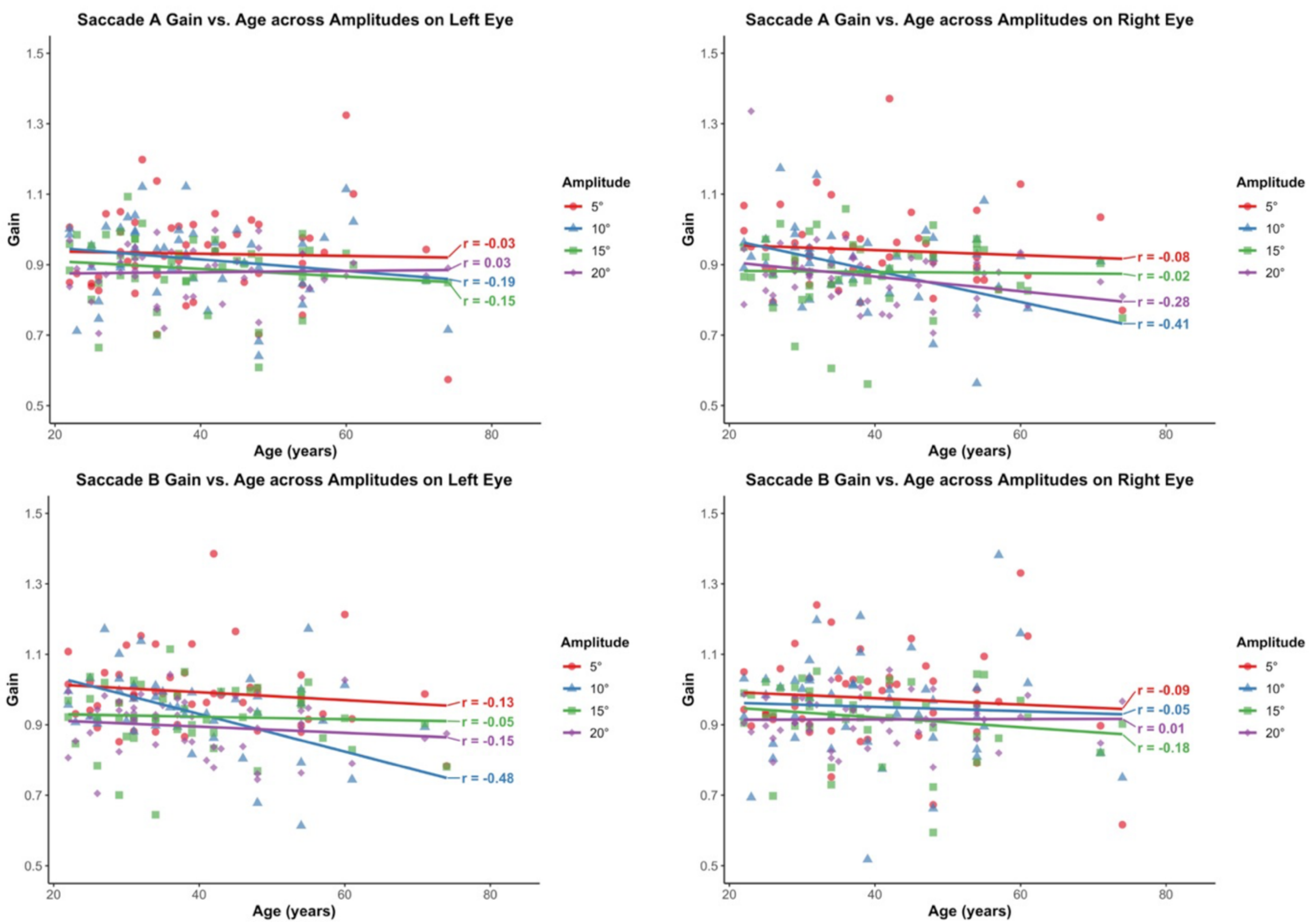

The left column shows data for the left eye, and the right column shows the right eye. Red circles and regression line present 5 °, blue triangles and regression line present saccade 10 °, green squares and regression line present 15 °, purple rhombuses and regression line present 20 °. “r” denotes the correlation coefficient in Pearson's correlation analysis.

Figure S10 The relationship between age and horizontal centripetal saccade gain in each amplitude (saccade C,D in Figure 1)

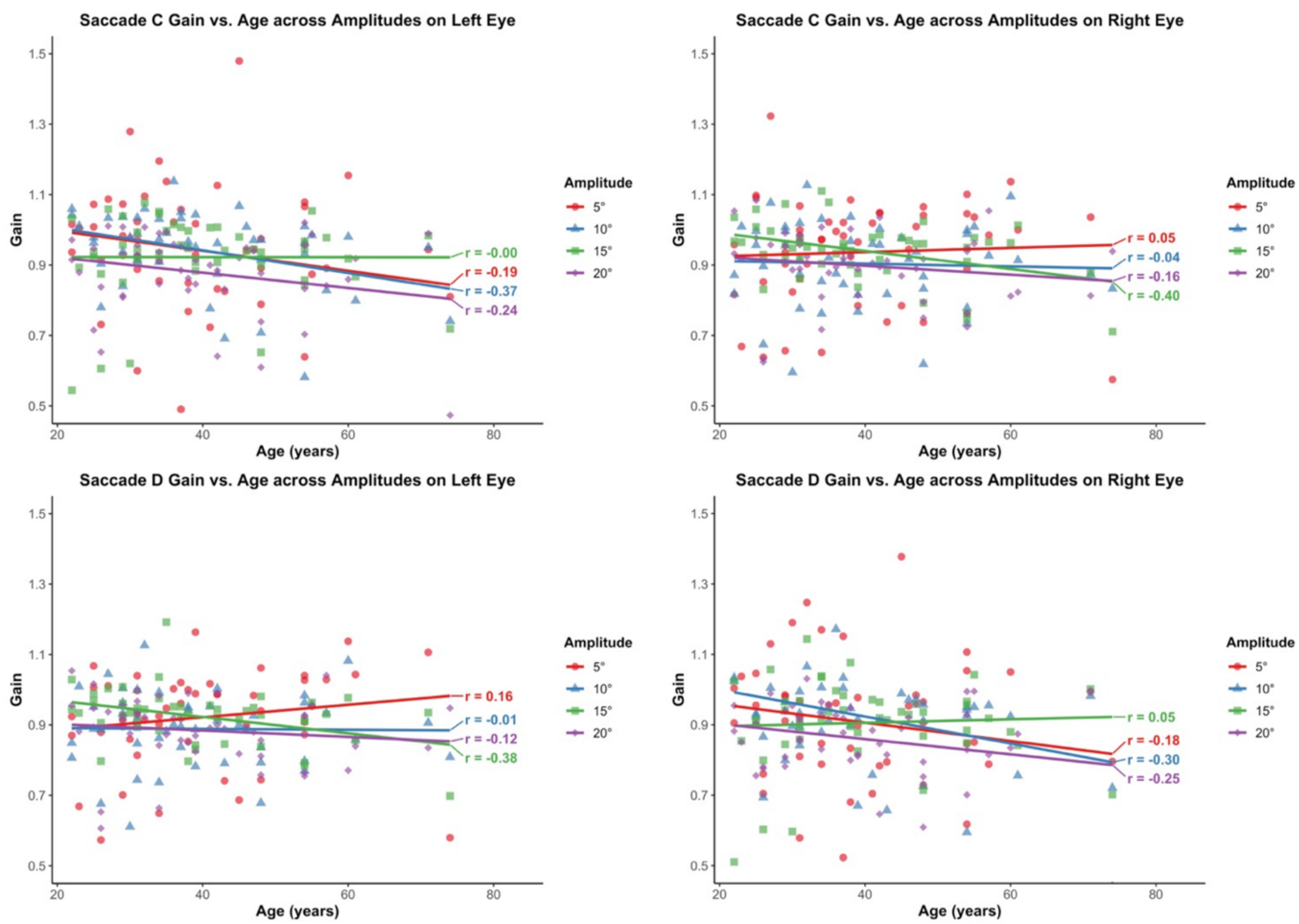

The left column shows data for the left eye, and the right column shows the right eye. Red circles and regression line present 5 °, blue triangles and regression line present saccade 10 °, green squares and regression line present 15 °, purple rhombuses and regression line present 20 °. "r" denotes the correlation coefficient in Pearson's correlation analysis.

Figure S11 The relationship between age and vertical centrifugal saccade velocity in each amplitude (saccade E,F in Figure 1)

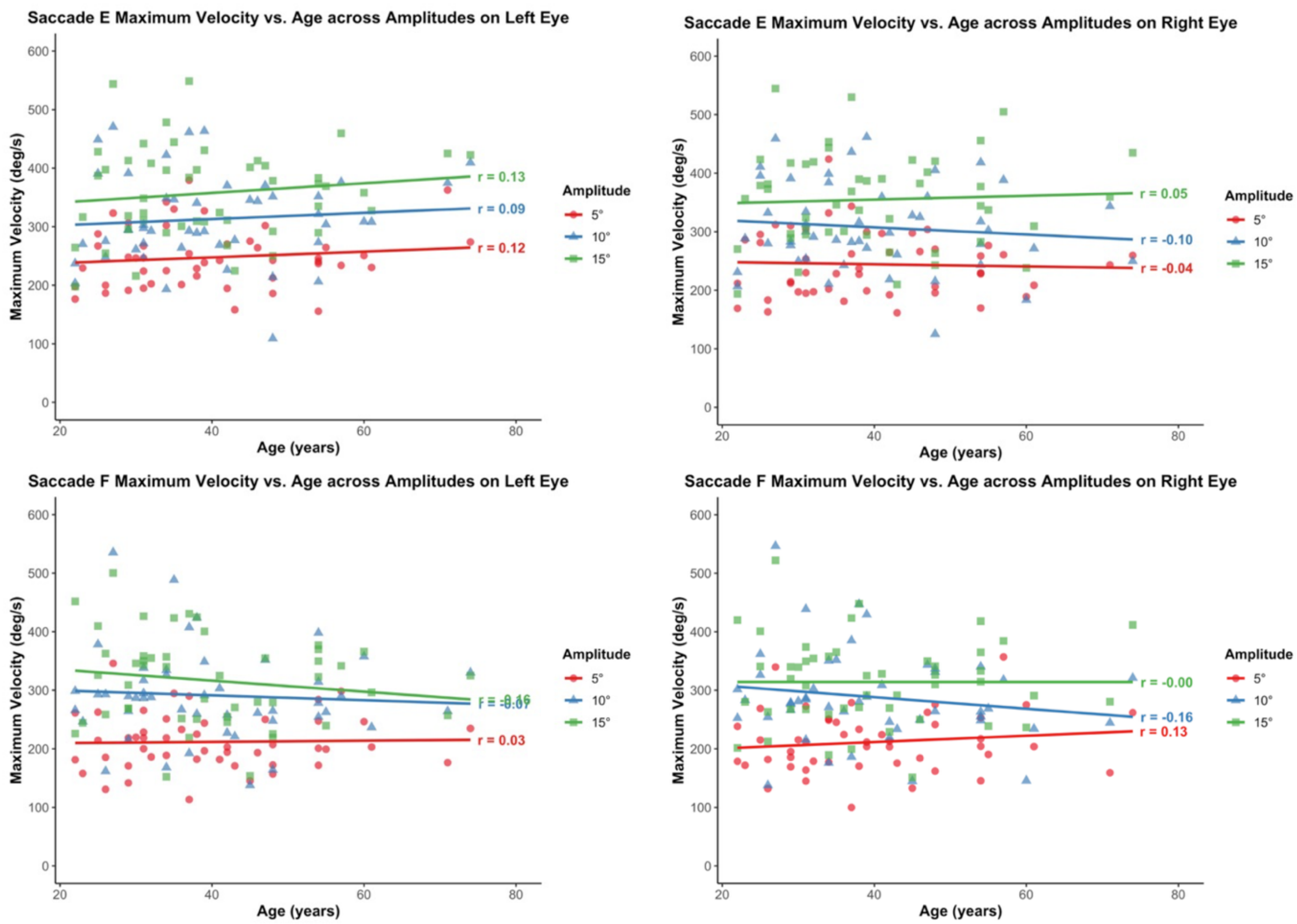

The left column shows data for the left eye, and the right column shows the right eye. Red circles and regression line present 5 °, blue triangles and regression line present saccade 10 °, green squares and regression line present 15 °. “r” denotes the correlation coefficient in Pearson's correlation analysis.

Figure S12 The relationship between age and vertical centripetal saccade velocity in each amplitude (saccade G,H in Figure 1)

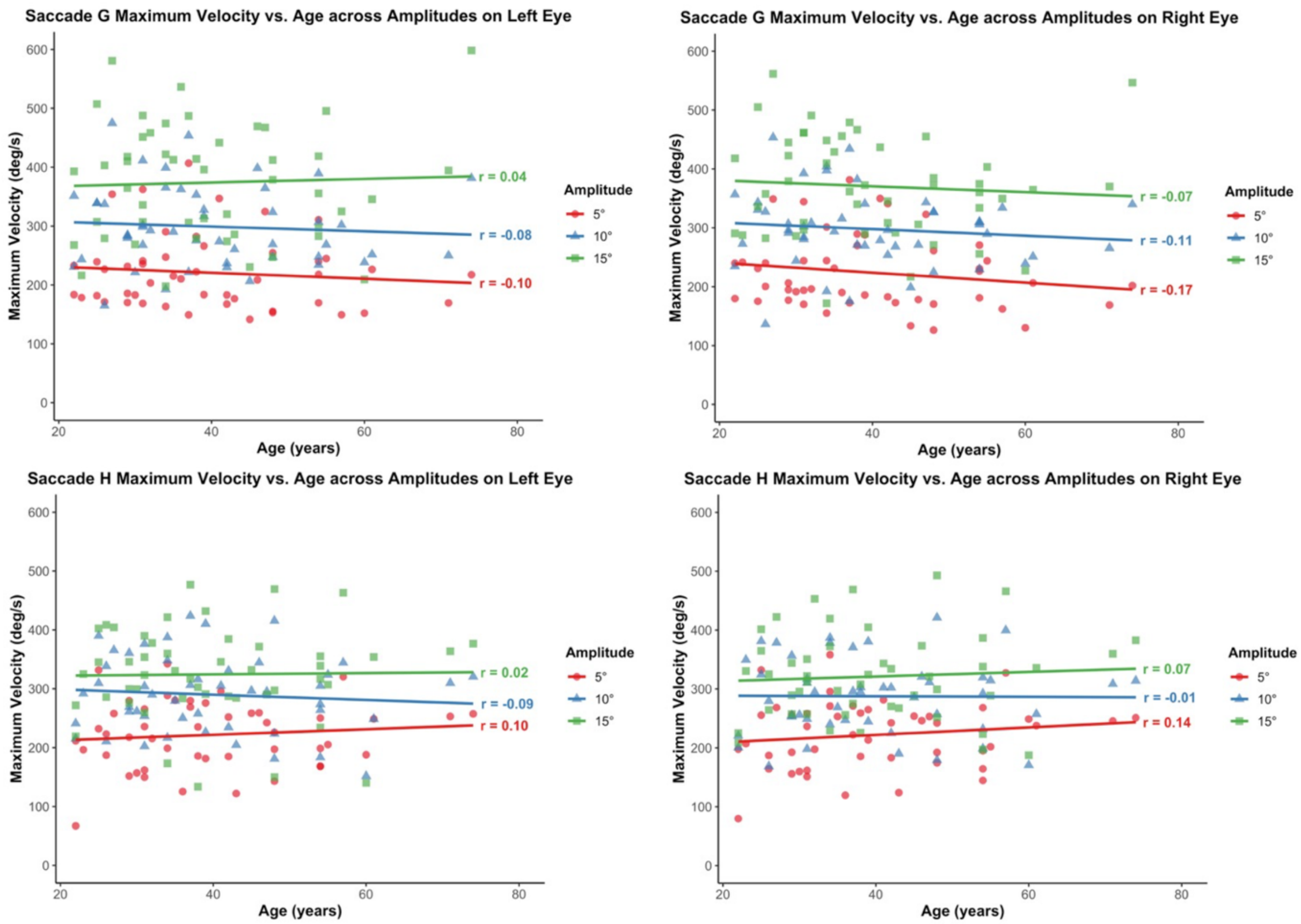

The left column shows data for the left eye, and the right column shows the right eye. Red circles and regression line present 5 °, blue triangles and regression line present saccade 10 °, green squares and regression line present 15 °. “r” denotes the correlation coefficient in Pearson's correlation analysis.

Figure S13 The relationship between age and vertical centrifugal saccade latency in each amplitude (saccade E,F in Figure 1)

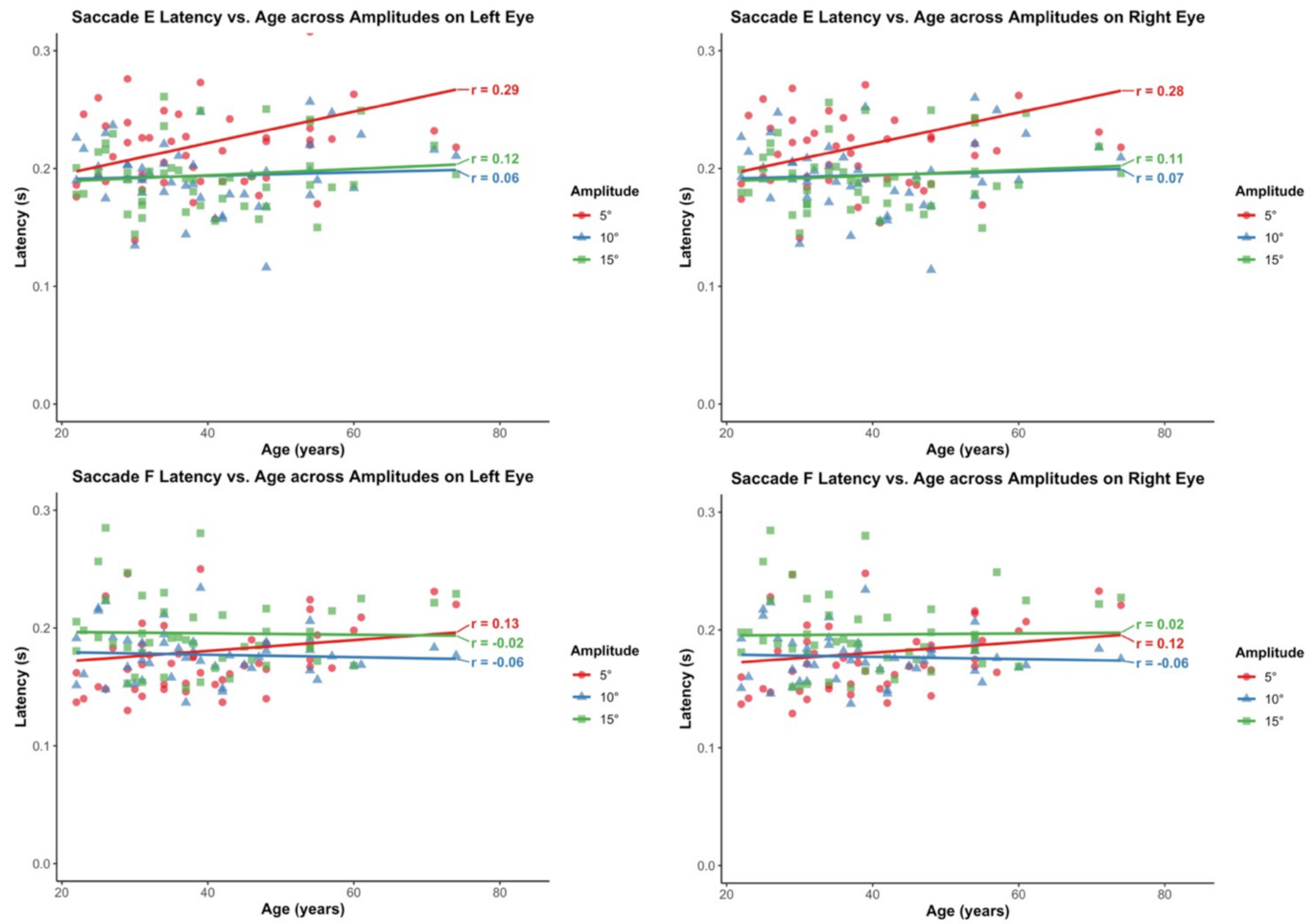

The left column shows data for the left eye, and the right column shows the right eye. Red circles and regression line present 5 °, blue triangles and regression line present saccade 10 °, green squares and regression line present 15 °. “r” denotes the correlation coefficient in Pearson's correlation analysis.

Figure S14 The relationship between age and vertical centripetal saccade latency in each amplitude (saccade G,H in Figure 1)

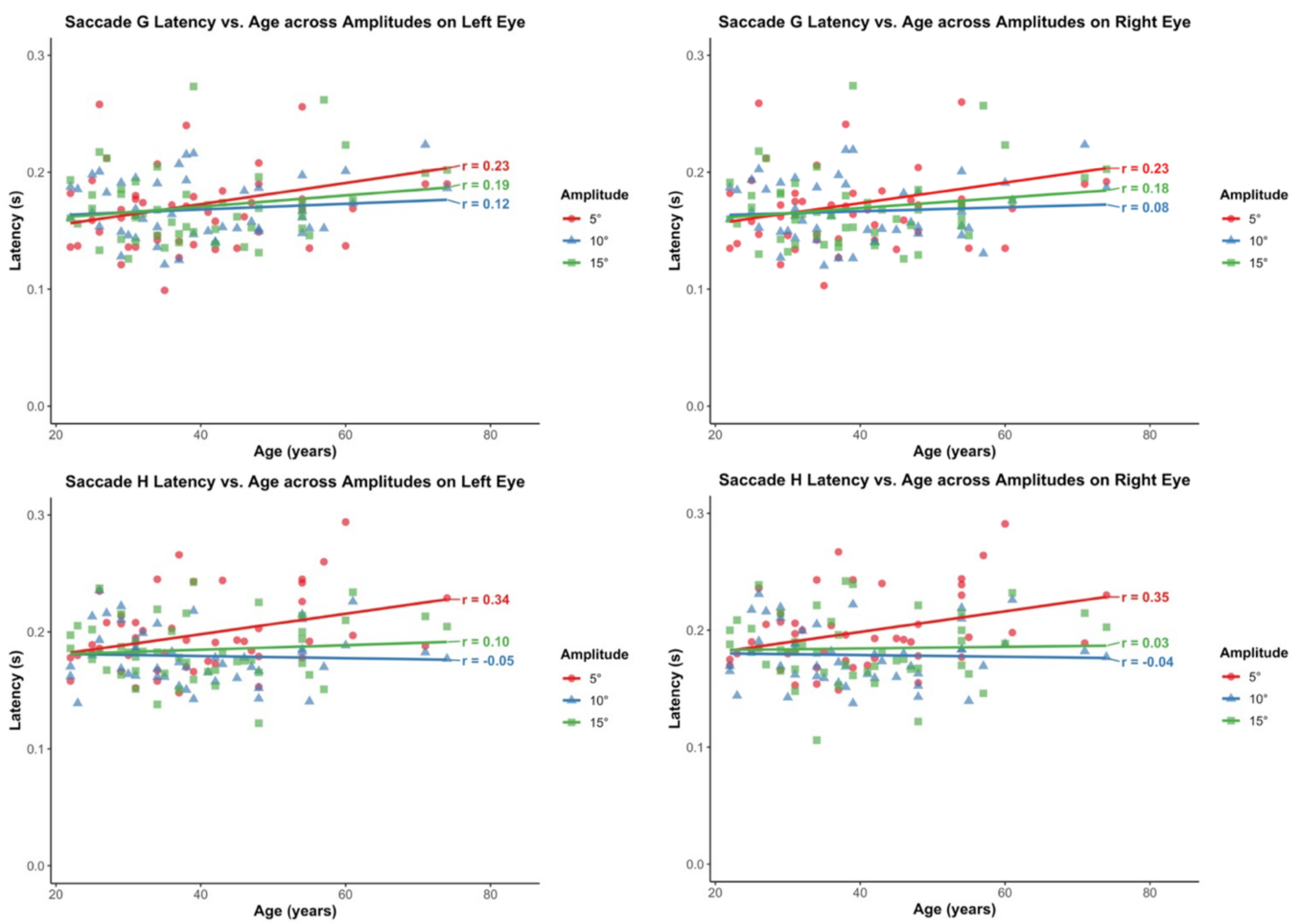

The left column shows data for the left eye, and the right column shows the right eye. Red circles and regression line present 5 °, blue triangles and regression line present saccade 10 °, green squares and regression line present 15 °. “r” denotes the correlation coefficient in Pearson's correlation analysis.

Figure S15 The relationship between age and vertical centrifugal saccade gain in each amplitude (saccade E,F in Figure 1)

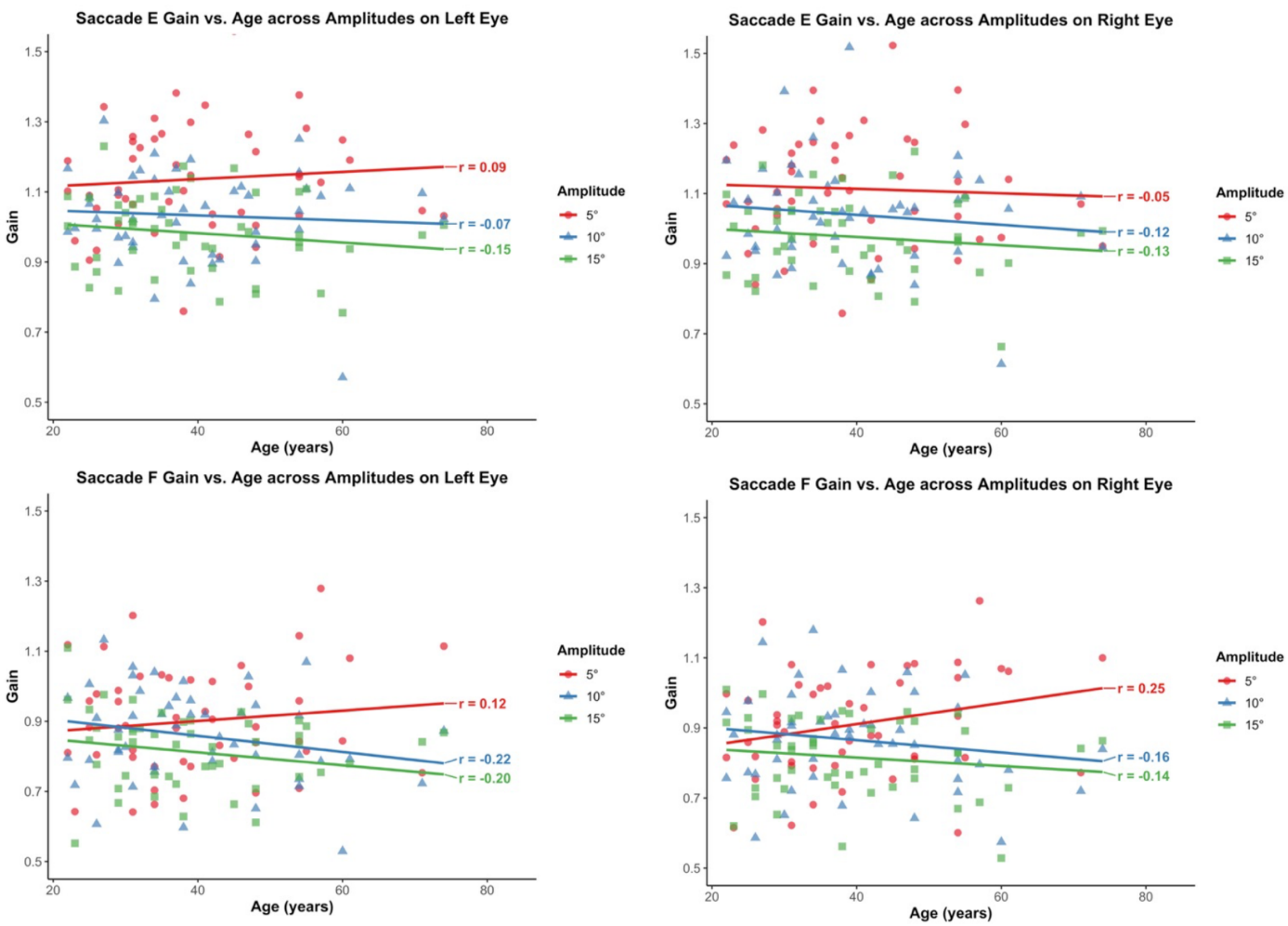

The left column shows data for the left eye, and the right column shows the right eye. Red circles and regression line present 5 °, blue triangles and regression line present saccade 10 °, green squares and regression line present 15 °. “r” denotes the correlation coefficient in Pearson's correlation analysis.

Figure S16 The relationship between age and vertical centripetal saccade gain in each amplitude (saccade G,H in Figure 1)

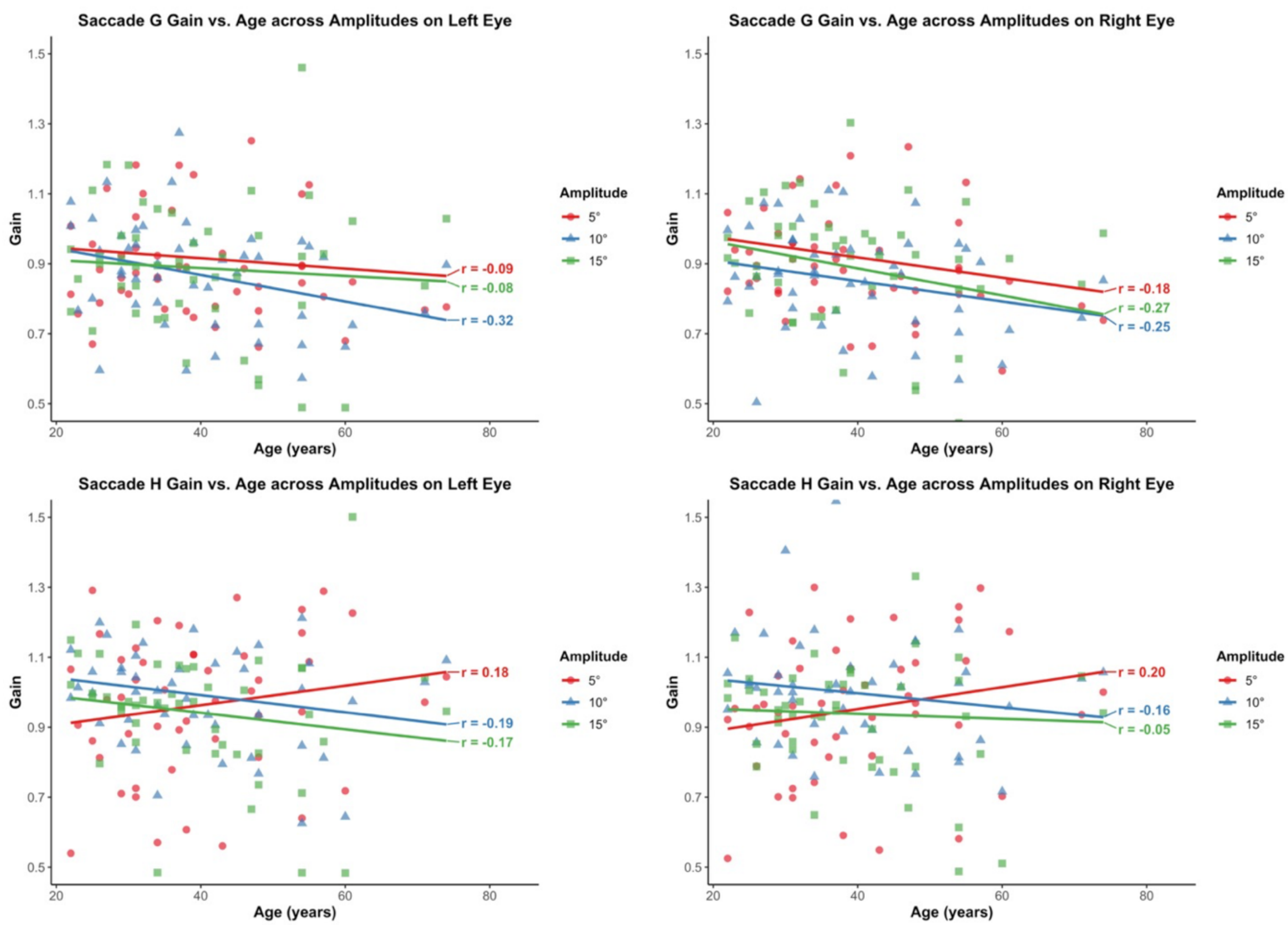

The left column shows data for the left eye, and the right column shows the right eye. Red circles and regression line present 5 °, blue triangles and regression line present saccade 10 °, green squares and regression line present 15 °. "r" denotes the correlation coefficient in Pearson's correlation analysis.

2 Supplementary Tables

Table S1 P-values and effect sizes for comparison of latency of horizontal saccades

5 degrees					
Eye	Comparison	Mean difference ($\bar{d}$)	95% CI	Effect size (d_z)	Adjusted p value
Lt eye	A vs B (B-A)	0.007	[-0.005, 0.019]	0.17	0.38
	A vs C (C-A)	-0.013	[-0.026, 0.000]	-0.30	0.20
	A vs D (D-A)	-0.011	[-0.021, -0.001]	-0.31	0.08
	B vs C (C-B)	-0.020	[-0.036, -0.004]	-0.37	0.06
	B vs D (D-B)	-0.018	[-0.031, -0.005]	-0.40	0.02
	C vs D (D-C)	0.002	[-0.010, 0.014]	0.05	0.98
Rt eye	A vs B (B-A)	-0.006	[-0.018, 0.006]	-0.15	0.60
	A vs C (C-A)	-0.018	[-0.030, -0.005]	-0.41	0.04
	A vs D (D-A)	-0.020	[-0.035, -0.005]	-0.38	0.06
	B vs C (C-B)	-0.012	[-0.021, -0.002]	-0.34	0.09
	B vs D (D-B)	-0.014	[-0.026, -0.001]	-0.31	0.12
	C vs D (D-C)	-0.002	[-0.014, 0.010]	-0.05	0.74

10 degrees					
Eye	Comparison	Mean difference ($\bar{d}$)	95% CI	Effect size (d_z)	Adjusted p value
Lt eye	A vs B (B-A)	0.001	[-0.011, 0.013]	0.02	0.91
	A vs C (C-A)	0.002	[-0.011, 0.014]	0.04	1
	A vs D (D-A)	-0.009	[-0.018, 0.001]	-0.26	0.15
	B vs C (C-B)	0.001	[-0.011, 0.013]	0.02	1
	B vs D (D-B)	-0.009	[-0.019, 0.000]	-0.30	0.16
	C vs D (D-C)	-0.010	[-0.021, 0.001]	-0.27	0.15
Rt eye	A vs B (B-A)	0.002	[-0.011, 0.014]	0.04	1
	A vs C (C-A)	-0.008	[-0.017, -0.001]	-0.27	0.35
	A vs D (D-A)	0.001	[-0.011, 0.014]	0.03	1
	B vs C (C-B)	-0.010	[-0.019, -0.001]	-0.32	0.20
	B vs D (D-B)	0.000	[-0.013, 0.013]	0.00	0.98
	C vs D (D-C)	0.010	[-0.001, 0.021]	0.26	0.32

15 degrees					
Eye	Comparison	Mean difference ($\bar{d}$)	95% CI	Effect size (d_z)	Adjusted p value
Lt eye	A vs B (B-A)	0.014	[0.005, 0.023]	0.43	0.03
	A vs C (C-A)	0.005	[-0.004, 0.014]	0.16	0.52
	A vs D (D-A)	0.006	[-0.003, 0.014]	0.19	0.59
	B vs C (C-B)	-0.009	[-0.016, -0.001]	-0.34	0.21
	B vs D (D-B)	-0.008	[-0.017, 0.001]	-0.25	0.24
	C vs D (D-C)	0.001	[-0.008, 0.010]	0.02	0.98
Rt eye	A vs B (B-A)	-0.012	[-0.021, -0.003]	-0.37	0.08
	A vs C (C-A)	-0.006	[-0.015, 0.002]	-0.22	0.54
	A vs D (D-A)	-0.009	[-0.017, -0.001]	-0.32	0.16
	B vs C (C-B)	0.005	[-0.003, 0.014]	-0.18	0.64
	B vs D (D-B)	0.003	[-0.007, 0.012]	0.09	1
	C vs D (D-C)	-0.003	[-0.011, 0.006]	-0.08	0.58

20 degrees					
Eye	Comparison	Mean difference ($\bar{d}$)	95% CI	Effect size (d_z)	Adjusted p value
Lt eye	A vs B (B-A)	0.020	[0.012, 0.028]	0.72	<0.01
	A vs C (C-A)	-0.005	[-0.013, 0.004]	-0.16	0.36
	A vs D (D-A)	-0.008	[-0.018, 0.001]	-0.25	0.24
	B vs C (C-B)	-0.025	[-0.033, -0.016]	-0.83	<0.01
	B vs D (D-B)	-0.028	[-0.040, -0.016]	-0.69	<0.01
	C vs D (D-C)	-0.004	[-0.013, 0.005]	-0.12	0.66
Rt eye	A vs B (B-A)	-0.018	[-0.026, -0.010]	-0.64	<0.01
	A vs C (C-A)	-0.027	[-0.039, -0.015]	-0.66	<0.01
	A vs D (D-A)	-0.024	[-0.032, -0.015]	-0.81	<0.01
	B vs C (C-B)	-0.009	[-0.018, 0.000]	-0.29	0.14
	B vs D (D-B)	-0.006	[-0.015, 0.002]	-0.21	0.30
	C vs D (D-C)	0.003	[-0.006, 0.012]	0.10	0.50

The letters A-D correspond to the directions A-D in Figure 1. The p-values were adjusted using the Bonferroni-Holm method (1 family = 6 paired test). Effect sizes are reported as Cohen's d_z for paired samples. CI: Confidence Intervals, *adjusted p value < 0.05.

Table S2 P-values and effect sizes for comparison of amplitude of horizontal saccades

5 degrees					
Eye	Comparison	Mean difference ($\bar{d}$)	95% CI	Effect size (d_z)	Adjusted p value
Lt eye	A vs B (B-A)	0.06	[0.03, 0.10]	0.51	<0.01
	A vs C (C-A)	0.01	[-0.05, 0.07]	0.05	0.73
	A vs D (D-A)	-0.01	[-0.05, 0.03]	-0.07	1
	B vs C (C-B)	-0.05	[-0.10, 0.00]	-0.30	0.16
	B vs D (D-B)	-0.07	[-0.12, -0.03]	-0.47	0.01
	C vs D (D-C)	-0.02	[-0.09, 0.05]	-0.08	1
Rt eye	A vs B (B-A)	0.03	[0.00, 0.07]	0.28	0.30
	A vs C (C-A)	0.00	[-0.05, 0.04]	-0.03	0.83
	A vs D (D-A)	-0.04	[-0.09, 0.01]	-0.20	0.49
	B vs C (C-B)	-0.04	[-0.08, 0.00]	-0.26	0.31
	B vs D (D-B)	-0.07	[-0.12, -0.01]	-0.36	0.10
	C vs D (D-C)	-0.03	[-0.10, 0.04]	-0.13	0.72

10 degrees					
Eye	Comparison	Mean difference ($\bar{\boldsymbol{d}}$)	95% CI	Effect size (d_z)	Adjusted p value
Lt eye	A vs B (B-A)	0.02	[-0.03, 0.06]	0.11	0.91
	A vs C (C-A)	0.03	[-0.01, 0.06]	0.20	0.50
	A vs D (D-A)	-0.03	[-0.06, 0.01]	-0.21	0.58
	B vs C (C-B)	0.01	[-0.03, 0.05]	0.08	0.59
	B vs D (D-B)	-0.04	[-0.08, 0.00]	-0.32	0.15
	C vs D (D-C)	-0.05	[-0.09, -0.02]	-0.43	0.03
Rt eye	A vs B (B-A)	0.07	[0.02, 0.12]	0.37	0.08
	A vs C (C-A)	0.02	[-0.02, 0.06]	0.17	0.77
	A vs D (D-A)	0.04	[-0.01, 0.09]	0.23	0.47
	B vs C (C-B)	-0.05	[-0.09, 0.00]	-0.32	0.16
	B vs D (D-B)	-0.03	[-0.09, 0.03]	-0.13	0.75
	C vs D (D-C)	0.02	[-0.03, 0.07]	0.13	0.39

15 degrees					
Eye	Comparison	Mean difference ($\bar{\boldsymbol{d}}$)	95% CI	Effect size (d_z)	Adjusted p value
Lt eye	A vs B (B-A)	0.03	[0.00, 0.07]	0.32	0.19
	A vs C (C-A)	0.03	[-0.01, 0.07]	0.25	0.38
	A vs D (D-A)	0.03	[0.00, 0.07]	0.32	0.17
	B vs C (C-B)	0.00	[-0.04, 0.04]	0.00	1
	B vs D (D-B)	0.00	[-0.03, 0.03]	0.00	0.99
	C vs D (D-C)	0.00	[-0.03, 0.03]	0.00	1
Rt eye	A vs B (B-A)	0.04	[0.01, 0.08]	0.36	0.08
	A vs C (C-A)	0.06	[0.03, 0.09]	0.52	<0.01
	A vs D (D-A)	0.03	[-0.01, 0.06]	0.21	0.48
	B vs C (C-B)	0.02	[-0.01, 0.05]	0.18	0.45
	B vs D (D-B)	-0.02	[-0.06, 0.02]	-0.12	0.43
	C vs D (D-C)	-0.03	[-0.07, 0.00]	-0.28	0.23

20 degrees					
Eye	Comparison	Mean difference ($\bar{\boldsymbol{d}}$)	95% CI	Effect size (d_z)	Adjusted p value
Lt eye	A vs B (B-A)	0.02	[-0.01, 0.05]	0.21	0.94
	A vs C (C-A)	0.00	[-0.03, 0.04]	0.02	0.91
	A vs D (D-A)	0.01	[-0.02, 0.03]	0.06	1
	B vs C (C-B)	-0.02	[0.05, 0.01]	-0.16	1
	B vs D (D-B)	-0.01	[-0.05, 0.02]	-0.12	1
	C vs D (D-C)	0.00	[-0.04, 0.04]	0.03	1
Rt eye	A vs B (B-A)	0.05	[0.02, 0.08]	0.45	0.02
	A vs C (C-A)	0.03	[0.00, 0.07]	0.26	0.25
	A vs D (D-A)	-0.01	[-0.04, 0.03]	-0.05	0.71
	B vs C (C-B)	-0.02	[-0.04, 0.01]	-0.19	0.41
	B vs D (D-B)	-0.06	[-0.09, -0.02]	-0.45	0.02
	C vs D (D-C)	-0.04	[-0.08, 0.00]	-0.28	0.22

The amplitude was expressed as gain (eye position) / (target position).

The letters A-D correspond to the directions A-D in Figure 1. The p-values were adjusted using the Bonferroni-Holm method (1 family = 6 paired test). Effect sizes are reported as Cohen's d_z for paired samples. CI: Confidence Intervals, *adjusted p value < 0.05.

Table S3 P-values and effect sizes for comparison of latency of vertical saccades

5 degrees					
Eye	Comparison	Mean difference ($\bar{d}$)	95% CI	Effect size (d_z)	Adjusted p value
Lt eye	E vs F (F-E)	-0.041	[-0.060, -0.021]	-0.61	<0.01
	E vs G (G-E)	-0.049	[-0.070, -0.027]	-0.66	<0.01
	E vs H (H-E)	-0.024	[-0.040, -0.007]	-0.41	0.02
	F vs G (G-F)	-0.008	[-0.025, 0.009]	-0.14	0.35
	H vs F (H-F)	0.017	[0.003, 0.032]	0.35	0.04
	G vs H (H-G)	0.025	[0.011, 0.039]	0.53	<0.01
Rt eye	E vs F (F-E)	-0.040	[-0.060, -0.021]	-0.60	<0.01
	E vs G (G-E)	-0.048	[-0.069, -0.026]	-0.64	<0.01
	E vs H (H-E)	-0.023	[-0.039, -0.006]	-0.39	0.03
	F vs G (G-F)	-0.007	[-0.024, 0.010]	-0.12	0.40
	F vs H (H-F)	0.018	[0.004, 0.032]	0.37	0.03
	G vs H (H-G)	0.025	[0.011, 0.038]	0.54	<0.01

10 degrees					
Eye	Comparison	Mean difference ($\bar{d}$)	95% CI	Effect size (d_z)	Adjusted p value
Lt eye	E vs F (F-E)	-0.016	[-0.024, -0.008]	-0.60	<0.01
	E vs G (G-E)	-0.026	[-0.034, -0.017]	-0.85	<0.01
	E vs H (H-E)	-0.014	[-0.022, -0.006]	-0.52	<0.01
	F vs G (G-F)	-0.009	[-0.016, -0.003]	-0.41	0.03
	H vs F (H-F)	0.002	[-0.006, 0.010]	0.08	0.60
	G vs H (H-G)	0.011	[0.003, 0.020]	0.38	0.02
Rt eye	E vs F (F-E)	-0.017	[-0.025, -0.009]	-0.61	<0.01
	E vs G (G-E)	-0.028	[-0.037, -0.018]	-0.85	<0.01
	E vs H (H-E)	-0.016	[-0.023, -0.008]	-0.57	<0.01
	F vs G (G-F)	-0.011	[-0.017, -0.004]	-0.46	<0.01
	F vs H (H-F)	0.002	[-0.006, 0.009]	0.06	0.68
	G vs H (H-G)	0.012	[0.004, 0.021]	0.42	0.01

15 degrees					
Eye	Comparison	Mean difference ($\bar{d}$)	95% CI	Effect size (d_z)	Adjusted p value
Lt eye	E vs F (F-E)	0.001	[-0.006, 0.009]	0.05	0.74
	E vs G (G-E)	-0.024	[-0.033, -0.014]	-0.71	<0.01
	E vs H (H-E)	-0.009	[-0.018, -0.001]	-0.34	0.07
	F vs G (G-F)	-0.025	[-0.035, -0.015]	-0.69	<0.01
	H vs F (H-F)	-0.011	[-0.020, -0.001]	-0.32	0.06
	G vs H (H-G)	0.014	[0.006, 0.023]	0.48	<0.01
Rt eye	E vs F (F-E)	0.002	[-0.006, 0.010]	0.08	0.59
	E vs G (G-E)	-0.025	[-0.034, -0.015]	-0.75	<0.01
	E vs H (H-E)	-0.010	[-0.018, -0.001]	-0.32	0.06
	F vs G (G-F)	-0.027	[-0.037, -0.017]	-0.78	<0.01
	F vs H (H-F)	-0.012	[-0.022, -0.001]	-0.33	0.08
	G vs H (H-G)	0.015	[0.006, 0.024]	0.46	<0.01

The letters E-H correspond to the E–H direction in Figure 1. The p-values were adjusted using the Bonferroni-Holm method (1 family = 6 paired test). Effect sizes are reported as Cohen's d_z for paired samples. CI: Confidence Intervals, *adjusted p value < 0.05.

Table S4 P-values and effect sizes for comparison of amplitude of vertical saccades

5 degrees					
Eye	Comparison	Mean difference ($\bar{\boldsymbol{d}}$)	95% CI	Effect size (d_z)	Adjusted p value
Lt eye	E vs F (F-E)	-0.24	[-0.29, -0.18]	-1.23	<0.01
	E vs G (G-E)	-0.22	[-0.28, -0.17]	-1.16	<0.01
	E vs H (H-E)	-0.17	[-0.23, -0.12]	-0.87	<0.01
	F vs G (G-F)	0.02	[-0.05, 0.08]	0.07	0.64
	H vs F (H-F)	0.06	[0.00, 0.12]	0.29	0.15
	G vs H (H-G)	0.05	[-0.04, 0.13]	0.17	0.52
Rt eye	E vs F (F-E)	-0.20	[-0.27, -0.14]	-0.87	<0.01
	E vs G (G-E)	-0.19	[-0.25, -0.14]	-0.99	<0.01
	E vs H (H-E)	-0.16	[-0.22, -0.11]	-0.84	<0.01
	F vs G (G-F)	0.01	[-0.06, 0.08]	0.03	0.82
	F vs H (H-F)	0.04	[-0.02, 0.11]	0.18	0.65
	G vs H (H-G)	0.03	[-0.05, 0.11]	0.12	0.83

10 degrees					
Eye	Comparison	Mean difference ($\bar{\boldsymbol{d}}$)	95% CI	Effect size (d_z)	Adjusted p value
Lt eye	E vs F (F-E)	-0.17	[-0.21, -0.13]	-1.24	<0.01
	E vs G (G-E)	-0.16	[-0.21, -0.12]	-1.02	<0.01
	E vs H (H-E)	-0.04	[-0.08, 0.00]	-0.27	0.13
	F vs G (G-F)	0.01	[-0.03, 0.05]	0.08	0.61
	H vs F (H-F)	0.13	[0.09, 0.18]	0.81	<0.01
	G vs H (H-G)	0.12	[0.07, 0.17]	0.70	<0.01
Rt eye	E vs F (F-E)	-0.17	[-0.23, -0.12]	-0.98	<0.01
	E vs G (G-E)	-0.19	[-0.24, -0.14]	-1.10	<0.01
	E vs H (H-E)	-0.04	[-0.08, 0.00]	-0.29	0.10
	F vs G (G-F)	-0.01	[-0.05, 0.02]	-0.11	0.43
	F vs H (H-F)	0.13	[0.08, 0.18]	0.76	<0.01
	G vs H (H-G)	0.15	[0.09, 0.20]	0.81	<0.01

15 degrees					
Eye	Comparison	Mean difference ($\bar{\boldsymbol{d}}$)	95% CI	Effect size (d_z)	Adjusted p value
Lt eye	E vs F (F-E)	-0.17	[-0.21, -0.13]	-1.37	<0.01
	E vs G (G-E)	-0.09	[-0.15, -0.04]	-0.49	<0.01
	E vs H (H-E)	-0.04	[-0.09, -0.01]	-0.23	0.24
	F vs G (G-F)	0.08	[0.02, 0.13]	0.39	0.03
	H vs F (H-F)	0.13	[0.08, 0.18]	0.69	<0.01
	G vs H (H-G)	0.05	[-0.02, 0.13]	0.22	0.14
Rt eye	E vs F (F-E)	-0.16	[-0.20, -0.12]	-1.24	<0.01
	E vs G (G-E)	-0.09	[-0.15, -0.03]	-0.45	<0.01
	E vs H (H-E)	-0.04	[-0.09, 0.02]	-0.20	0.18
	F vs G (G-F)	0.07	[0.02, 0.12]	0.43	0.01
	F vs H (H-F)	0.12	[0.06, 0.18]	0.60	<0.01
	G vs H (H-G)	0.05	[-0.02 0.12]	0.21	0.29

The amplitude was expressed as gain (eye position) / (target position).

The letters E-H correspond to the E–H direction in Figure 1. The p-values were adjusted using the Bonferroni-Holm method (1 family = 6 paired test). Effect sizes are reported as Cohen's d_z for paired samples. CI: Confidence Intervals, *adjusted p value < 0.05.

Table S5 The Pearson's correlation analysis of horizontal saccades

Maximum velocity		A	B	C	D
Left eye	20 degrees	0.03 (0.82)	-0.16 (0.28)	-0.15 (0.30)	-0.06 (0.68)
	15 degrees	-0.05 (0.72)	-0.05 (0.74)	0.02 (0.90)	-0.06 (0.69)
	10 degrees	0.08 (0.60)	-0.18 (0.21)	-0.01 (0.94)	0.11 (0.47)
	5 degrees	0.10 (0.50)	0.07 (0.63)	0.06 (0.69)	0.24 (0.11)
Right eye	20 degrees	-0.07 (0.63)	-0.08 (0.60)	-0.10 (0.48)	-0.11 (0.46)
	15 degrees	-0.01 (0.95)	-0.04 (0.80)	-0.12 (0.42)	0.04 (0.80)
	10 degrees	-0.17 (0.24)	0.09 (0.54)	0.07 (0.64)	0.02 (0.88)
	5 degrees	0.05 (0.73)	0.05 (0.74)	0.16 (0.27)	-0.00 (1.00)
Latency		A	B	C	D
Left eye	20 degrees	0.17 (0.26)	-0.19 (0.19)	-0.05 (0.74)	-0.16 (0.29)
	15 degrees	-0.12 (0.42)	-0.00 (0.97)	0.04 (0.81)	0.05 (0.76)
	10 degrees	-0.09 (0.54)	-0.04 (0.79)	0.06 (0.68)	-0.06 (0.67)
	5 degrees	0.05 (0.76)	0.06 (0.68)	-0.05 (0.74)	0.17 (0.24)
Right eye	20 degrees	-0.19 (0.20)	0.15 (0.32)	-0.12 (0.43)	-0.05 (0.76)
	15 degrees	-0.00 (0.98)	-0.13 (0.37)	0.07 (0.65)	0.05 (0.74)
	10 degrees	-0.04 (0.81)	-0.07 (0.62)	-0.07 (0.63)	0.05 (0.73)
	5 degrees	0.07 (0.62)	0.05 (0.73)	0.17 (0.25)	-0.05 (0.75)
Gain		A	B	C	D
Left eye	20 degrees	0.03 (0.82)	-0.15 (0.32)	-0.24 (0.10)	-0.12 (0.41)
	15 degrees	-0.15 (0.32)	-0.05 (0.72)	-0.00 (0.99)	-0.38 (<0.01*)
	10 degrees	-0.19 (0.20)	-0.48 (<0.01)	-0.37 (0.01*)	-0.01 (0.92)

	5 degrees	-0.03 (0.84)	-0.13 (0.37)	-0.19 (0.20)	0.16 (0.27)
Right eye	20 degrees	-0.28 (0.05)	0.01 (0.95)	-0.16 (0.28)	-0.25 (0.09)
	15 degrees	-0.02 (0.89)	-0.18 (0.22)	-0.40 (<0.01*)	0.05 (0.73)
	10 degrees	-0.41 (<0.01*)	-0.05 (0.73)	-0.04 (0.78)	-0.30 (0.04*)
	5 degrees	-0.08 (0.57)	-0.09 (0.56)	0.05 (0.74)	-0.18 (0.23)

The letters A-D correspond to the directions A-D in Figure 1.

Data are shown as correlation coefficients (p-values). *adjusted p value < 0.05.

Table S6 The Pearson's correlation analysis of vertical saccades

Maximum velocity		E	F	G	H
Left eye	15 degrees	0.13 (0.37)	-0.16 (0.29)	0.04 (0.79)	0.02 (0.91)
	10 degrees	0.09 (0.55)	-0.07 (0.64)	-0.08 (0.61)	-0.09 (0.56)
	5 degrees	0.12 (0.41)	0.03 (0.85)	-0.10 (0.49)	0.10 (0.48)
Right eye	15 degrees	0.05(0.73)	-0.00 (1.00)	-0.07 (0.62)	0.07 (0.65)
	10 degrees	-0.10(0.48)	-0.16 (0.28)	-0.11 (0.45)	-0.01 (0.95)
	5 degrees	-0.04(0.77)	0.13 (0.37)	-0.17 (0.25)	0.14 (0.34)
**Latency**		**E**	**F**	**G**	**H**
Left eye	15 degrees	0.12 (0.43)	-0.02 (0.88)	0.19 (0.19)	0.10 (0.52)
	10 degrees	0.06 (0.66)	-0.06 (0.67)	0.12 (0.41)	-0.05 (0.75)
	5 degrees	0.29 (0.05*)	0.13 (0.39)	0.23 (0.11)	0.34 (0.02*)
Right eye	15 degrees	0.11 (0.46)	0.02 (0.91)	0.18 (0.22)	0.03 (0.83)
	10 degrees	0.07 (0.66)	-0.06 (0.69)	0.08 (0.58)	-0.04 (0.80)
	5 degrees	0.28 (0.05)	0.12 (0.40)	0.23 (0.12)	0.35 (0.02*)
**Gain**		**E**	**F**	**G**	**H**
Left eye	15 degrees	-0.15 (0.30)	-0.20 (0.18)	-0.08 (0.61)	-0.17 (0.26)
	10 degrees	-0.07 (0.64)	-0.22 (0.13)	-0.32 (0.03*)	-0.19 (0.21)
	5 degrees	0.09 (0.56)	0.12 (0.41)	-0.09 (0.54)	0.18 (0.23)
Right eye	15 degrees	-0.13 (0.37)	-0.14 (0.35)	-0.27 (0.06)	-0.05 (0.75)
	10 degrees	-0.12 (0.41)	-0.16 (0.26)	-0.25 (0.08)	-0.16 (0.27)
	5 degrees	-0.05 (0.74)	0.25 (0.08)	-0.18 (0.23)	0.20 (0.17)

The letters E-H correspond to the E–H direction in Figure 1.

Data are shown as correlation coefficients (p-values). *adjusted p value < 0.05.

Table S7 Gain adjusted maximum velocities of horizontal saccades in 4 directions

Maximum velocity / gain (deg/sec)		A	B	C	D
Left eye	20 degrees	419.19 (63.70)	432.27 (59.66)	481.08 (102.30)	490.70 (70.31)
	15 degrees	383.49 (56.73)	388.61 (58.62)	425.50 (84.12)	432.22 (72.53)
	10 degrees	334.96 (57.86)	348.73 (127.57)	350.24 (59.14)	370.52 (55.02)
	5 degrees	255.74 (33.91)	245.43 (38.13)	251.34 (54.00)	259.00 (53.88)
Right eye	20 degrees	432.99 (70.74)	416.48 (61.97)	450.35 (58.72)	523.17 (120.27)
	15 degrees	402.90 (85.91)	377.81 (52.29)	403.83 (69.76)	452.73 (104.36)
	10 degrees	357.12 (117.12)	320.56 (55.00)	347.22 (53.06)	372.06 (64.24)
	5 degrees	256.21 (39.26)	243.12 (33.69)	242.38 (49.06)	264.75 (50.45)

The letters A-D correspond to the directions A-D in Figure 1. The gain adjusted maximum velocity was calculated by (actual maximum velocity) / (saccade gain).

Data are shown as the mean (SD).

Table S8 P-values and effect sizes for comparison of gain adjusted maximum velocity of horizontal saccades

5 degrees					
Eye	Comparison	Mean difference ($\bar{\boldsymbol{d}}$)	95% CI	Effect size (d_z)	Adjusted p value
Lt eye	A vs B (B-A)	-10.30	[-18.57, -2.04]	-0.36	0.09
	A vs C (C-A)	-6.13	[-18.20, 5.94]	-0.15	1
	A vs D (D-A)	4.82	[-8.02, 17.66]	0.11	0.60
	B vs C (C-B)	4.17	[-6.45, 14.79]	0.11	1
	B vs D (D-B)	15.12	[2.31, 27.93]	0.34	0.18
	C vs D (D-C)	10.95	[-5.32, 27.22]	0.20	1
Rt eye	A vs B (B-A)	-13.09	[-20.39, -5.79]	-0.52	<0.01*
	A vs C (C-A)	-13.83	[-24.42, -3.24]	-0.38	0.03*
	A vs D (D-A)	8.54	[-2.70, 19.79]	0.22	0.26
	B vs C (C-B)	-0.74	[-11.51, 10.03]	-0.02	0.89
	B vs D (D-B)	21.63	[9.06, 34.20]	0.50	<0.01*
	C vs D (D-C)	22.37	[5.54, 39.20]	0.39	0.04*

10 degrees					
Eye	Comparison	Mean difference ($\bar{d}$)	95% CI	Effect size (d_z)	Adjusted p value
Lt eye	A vs B (B-A)	13.76	[-17.83, 45.36]	0.13	0.77
	A vs C (C-A)	17.08	[0.72, 33.43]	0.30	0.23
	A vs D (D-A)	35.11	[20.44, 49.79]	0.69	<0.01*
	B vs C (C-B)	3.31	[-26.94, 33.57]	0.03	0.92
	B vs D (D-B)	21.35	[-13.51, 56.22]	0.18	0.65
	C vs D (D-C)	18.04	[3.70, 32.38]	0.37	0.03*
Rt eye	A vs B (B-A)	-36.56	[-63.40, -9.72]	-0.40	0.03*
	A vs C (C-A)	-9.90	[-41.22, 21.42]	-0.09	0.53
	A vs D (D-A)	14.94	[-13.13, 43.01]	0.15	0.58
	B vs C (C-B)	26.66	[11.17, 42.16]	0.50	<0.01*
	B vs D (D-B)	51.50	[37.24, 65.76]	1.05	<0.01*
	C vs D (D-C)	24.84	[10.82, 38.85]	0.51	<0.01*

15 degrees					
Eye	Comparison	Mean difference ($\bar{d}$)	95% CI	Effect size (d_z)	Adjusted p value
Lt eye	A vs B (B-A)	5.12	[-7.61, 17.85]	0.12	0.85
	A vs C (C-A)	42.30	[15.58, 69.02]	0.46	0.01*
	A vs D (D-A)	50.09	[31.02, 69.15]	0.76	<0.01*
	B vs C (C-B)	37.18	[13.51, 60.85]	0.46	<0.01*
	B vs D (D-B)	44.97	[28.26, 61.68]	0.78	<0.01*
	C vs D (D-C)	7.79	[-19.36, 34.93]	0.08	0.62
Rt eye	A vs B (B-A)	-25.10	[-46.33, -3.87]	-0.34	0.04*
	A vs C (C-A)	0.93	[-21.49, 23.35]	0.01	0.93
	A vs D (D-A)	49.83	[17.27, 82.39]	0.44	0.01*
	B vs C (C-B)	26.03	[9.44, 42.62]	0.46	0.01*
	B vs D (D-B)	74.93	[42.63, 107.22]	0.67	<0.01*
	C vs D (D-C)	48.90	[17.83, 79.97]	0.46	0.01*

20 degrees					
Eye	Comparison	Mean difference ($\bar{\boldsymbol{d}}$)	95% CI	Effect size (d_z)	Adjusted p value
Lt eye	A vs B (B-A)	13.08	[-0.52, 26.68]	0.28	0.12
	A vs C (C-A)	49.97	[15.68, 84.25]	0.42	<0.01*
	A vs D (D-A)	59.72	[31.48, 87.97]	0.61	<0.01*
	B vs C (C-B)	36.88	[5.06, 68.71]	0.34	<0.01*
	B vs D (D-B)	46.64	[15.61, 77.67]	0.44	<0.01*
	C vs D (D-C)	9.76	[-21.62, 41.14]	0.09	0.54
Rt eye	A vs B (B-A)	-16.51	[-30.93, -2.09]	-0.33	0.05
	A vs C (C-A)	17.36	[-0.37, 35.09]	0.28	0.05
	A vs D (D-A)	90.18	[63.29, 117.07]	0.97	<0.01*
	B vs C (C-B)	33.87	[21.26, 46.48]	0.78	<0.01*
	B vs D (D-B)	106.69	[78.05, 135.32]	1.08	<0.01*
	C vs D (D-C)	72.82	[40.24, 105.39]	0.65	<0.01*

The letters A-D correspond to the directions A-D in Figure 1. The p-values were adjusted using the Bonferroni-Holm method (1 family = 6 paired test). Effect sizes are reported as Cohen's d_z for paired samples. CI: Confidence Intervals, *adjusted p value < 0.05.

Table S9 Gain adjusted maximum velocities of vertical saccades in 4 directions

Maximum velocity / gain (deg/sec)		E	F	G	H
Left eye	15 degrees	366.60 (69.44)	397.12 (81.74)	442.16 (122.09)	358.93 (72.09)
	10 degrees	302.16 (64.72)	341.45 (81.96)	358.57 (76.68)	296.67 (52.79)
	5 degrees	222.79 (40.04)	237.06 (47.39)	243.27 (44.35)	229.38 (43.92)
Right eye	15 degrees	366.25 (70.29)	392.22 (86.42)	447.42 (130.51)	354.16 (74.56)
	10 degrees	297.71 (65.52)	334.44 (80.80)	360.52 (78.15)	296.26 (57.23)
	5 degrees	222.95 (44.99)	232.31 (44.61)	268.82 (196.20)	233.19 (46.94)

The letters E-H correspond to the E–H direction in Fig. 1. The gain adjusted maximum velocity was calculated by (actual maximum velocity) / (saccade gain).

Data are shown as the mean (SD).

Table S10 P-values and effect sizes for comparison of gain adjusted maximum velocity of vertical saccades

5 degrees					
Eye	Comparison	Mean difference ($\bar{d}$)	95% CI	Effect size (d_z)	Adjusted p value
Lt eye	E vs F (F-E)	14.27	[-1.47, 30.02]	0.26	0.30
	E vs G (G-E)	20.47	[7.64, 33.31]	0.46	0.01*
	E vs H (H-E)	6.59	[-4.20, 17.38]	0.18	0.68
	F vs G (G-F)	6.20	[-5.44, 17.85]	0.15	0.58
	H vs F (H-F)	-7.68	[-23.68, 8.32]	-0.14	0.34
	G vs H (H-G)	-13.88	[-28.45, 0.69]	-0.28	0.31
Rt eye	E vs F (F-E)	9.36	[-4.96, 23.68]	0.19	0.78
	E vs G (G-E)	45.87	[-6.09, 97.83]	0.26	0.49
	E vs H (H-E)	10.24	[-2.11, 22.59]	0.24	0.51
	F vs G (G-F)	36.51	[-19.73, 92.74]	0.19	0.59
	F vs H (H-F)	0.88	[-13.02, 14.78]	0.02	0.90
	G vs H (H-G)	-35.63	[-91.77, 20.52]	-0.18	0.42

10 degrees					
Eye	Comparison	Mean difference ($\bar{\boldsymbol{d}}$)	95% CI	Effect size (d_z)	Adjusted p value
Lt eye	E vs F (F-E)	39.29	[12.70, 65.89]	0.43	0.01*
	E vs G (G-E)	56.41	[32.92, 79.91]	0.70	<0.01*
	E vs H (H-E)	-5.49	[-19.98, 9.00]	-0.11	0.45
	F vs G (G-F)	17.12	[-1.14, 35.38]	0.27	0.13
	H vs F (H-F)	-44.78	[-66.92, -22.65]	-0.59	<0.01*
	G vs H (H-G)	-61.90	[-83.28, -40.53]	-0.84	<0.01*
Rt eye	E vs F (F-E)	36.73	[14.15, 59.31]	0.47	<0.01*
	E vs G (G-E)	62.82	[39.87, 85.77]	0.79	<0.01*
	E vs H (H-E)	-1.45	[-15.30, 12.41]	-0.03	0.83
	F vs G (G-F)	26.09	[5.22, 46.95]	0.36	0.03*
	F vs H (H-F)	-38.18	[-59.28, -17.07]	-0.53	<0.01*
	G vs H (H-G)	-64.26	[-86.40, -42.12]	-0.84	<0.01*

15 degrees					
Eye	Comparison	Mean difference ($\bar{d}$)	95% CI	Effect size (d_z)	Adjusted p value
Lt eye	E vs F (F-E)	30.52	[5.04, 56.00]	0.35	0.04*
	E vs G (G-E)	75.56	[42.21, 108.91]	0.66	<0.01*
	E vs H (H-E)	-7.67	[-27.24, 11.90]	-0.11	0.43
	F vs G (G-F)	45.04	[9.41, 80.67]	0.37	0.04*
	H vs F (H-F)	-38.19	[-64.83, -11.55]	-0.42	0.02*
	G vs H (H-G)	-83.23	[-122.50, -43.97]	-0.62	<0.01*
Rt eye	E vs F (F-E)	25.96	[-1.77, 53.69]	0.27	0.13
	E vs G (G-E)	81.16	[45.26, 117.06]	0.66	<0.01*
	E vs H (H-E)	-12.10	[-29.59, 5.39]	-0.20	0.17
	F vs G (G-F)	55.20	[25.63, 84.78]	0.54	<0.01*
	F vs H (H-F)	-38.06	[-65.26, -10.85]	-0.41	0.02*
	G vs H (H-G)	-93.26	[-130.58, -55.94]	-0.73	<0.01*

The letters E-H correspond to the E–H direction in Figure 1. The p-values were adjusted using the Bonferroni-Holm method (1 family = 6 paired test). Effect sizes are reported as Cohen's d_z for paired samples. CI: Confidence Intervals, *adjusted p value < 0.05.
