# Supplementary material for: Directional differences in saccades
Source: Front Neurol. 2026 Oct 23;17:1945647. doi: 10.3389/fneur.2026.1945647 (PMC13741797; doi:10.3389/fneur.2026.1945647)
Supplement: Supplementary file 2 [file Supplementary_file_1.docx]

Supplementary Material

# 1 Supplementary text file

# The frictional force between objects is proportional to the contact area when the pressure per unit area between the objects is the same. By applying this concept to the contact surface between the eyeball and eyelid, when the eyeball slides into contact with the eyelid, its velocity is considered to depend on the contact area between the eyeball and eyelid. When the eyeball is in the upper hemifield (looking upward), the upper eyelid is elevated, and thus, the eyelid coverage rate of the eyeball decreases, whereas when the eyeball is in the lower hemifield (looking downward), the upper eyelid droops, and thus, the eyelid coverage rate increases. Higher eyelid coverage is expected to increase the contact area with the tissues around the eyeball, thereby increasing friction. This increased friction may slow down the saccade velocity within the lower hemifield.

# Highly consistent with this theoretical relationship between contact area, friction, and velocity, previous physical and biotribological assessments of the ocular surface have confirmed that the coefficient of friction directly determines sliding kinetics between the eyelid and the globe (1). However, saccades involve much smaller eyelid movements than blinking and are driven by different neural pathways and velocity sequences; whether physical friction between the eyelid and the globe impacts saccadic dynamics remains unexamined.

# Thus, for reference, based on the above biotribological principles, additional experiments were conducted to investigate the effect of eyelid coverage rate on saccadic velocity during vertical gaze shifts on some of the participants in this study. Seventeen subjects (mean [SD] age = 43.0 [11.8] y, male ratio = 52.9%) who participated in this study performed a 15 ° vertical saccade task, and their right eye fissures were photographed from the frontal direction of the eyeball during 15-degree upward and 15-degree downward gazing. VOR-3 Frenzel goggles with a CCD camera at a sampling rate of 60 Hz (provided by Morita Co., Ltd., Tokyo, Japan), different from the ones used in this study, were used for imaging. The contact area between the eyeball and surrounding tissue was calculated by approximating the human eyeball to a sphere with a diameter of 23 mm, which is the average size of the human eyeball (2), and subtracting the palpebral fissure area from its surface area. The relationship between eye-eyelid contact area and saccade velocity for the seventeen subjects in this study is shown in the following Table.

In this study, the ratio of the contact area of the eyeball with the surrounding tissue when looking 15 °up and 15 °down was approximately consistent with the ratio of the velocities of a 15 °up saccade and a 15 °down saccade. This result suggests that frictional resistance with the surrounding tissues of the eyeball, which changes depending on eyelid position, may be one reason for the difference in velocity between vertical saccades moving within the upper and those moving within the lower hemifields.

Because friction between the eyeball and eyelid cannot be directly measured using the methods available in this study, we indirectly estimated frictional resistance by measuring the contact area between the eyelid and the globe. To more rigorously test this hypothesis, future studies should directly measure the frictional force between the eyeball and eyelid in the upper and lower hemifields during saccades and further examine its relationship with saccade velocity.

# 2 Supplementary Tables

| Eye position | 15 degrees upper | 15 degrees lower | Area ratio of upper and lower positions |
| --- | --- | --- | --- |
| Covered area  (mm^2^) | 1413.587 | 1470.335 | 0.961 |
|  |  |  |  |
| Moving position | 0° to upward 15° saccade | 0° to downward 15° saccade | Velocity ratio of upward and downward saccades |
| Saccade velocity  (deg/sec) | 351.40 | 308.90 | 0.879 |

The relationship between eyeball area covered by surrounding tissue and centrifugal saccade velocity by eye position

References

[1] Pult H, Tosatti SGP, Spencer ND, Asfour JM, Ebenhoch M, Murphy PJ. Spontaneous Blinking from a Tribological Viewpoint. Ocul Surf (2015) 13(3):236-49. doi: 10.1016/j.jtos.2014.12.004.

[2] Bekerman I, Gottlieb P, Vaiman M. Variations in Eyeball Diameters of the Healthy Adults. J Ophthalmol 2014:2014:503645 doi: 10.1155/2014/503645.
